# BeWith: A Between-Within method to discover relationships between cancer modules via integrated analysis of mutual exclusivity, co-occurrence and functional interactions

**DOI:** 10.1371/journal.pcbi.1005695

**Published:** 2017-10-12

**Authors:** Phuong Dao, Yoo-Ah Kim, Damian Wojtowicz, Sanna Madan, Roded Sharan, Teresa M. Przytycka

**Affiliations:** 1 National Center of Biotechnology Information, National Library of Medicine, NIH, Bethesda, MD, United States of America; 2 Department of Computer Science, University of Maryland, College Park, MD, United States of America; 3 Blavatnik School of Computer Science, Tel Aviv University, Tel Aviv, Israel; Johns Hopkins University, UNITED STATES

## Abstract

The analysis of the mutational landscape of cancer, including mutual exclusivity and co-occurrence of mutations, has been instrumental in studying the disease. We hypothesized that exploring the interplay between co-occurrence, mutual exclusivity, and functional interactions between genes will further improve our understanding of the disease and help to uncover new relations between cancer driving genes and pathways. To this end, we designed a general framework, BeWith, for identifying modules with different combinations of mutation and interaction patterns. We focused on three different settings of the BeWith schema: (i) BeME-WithFun, in which the relations between modules are enriched with mutual exclusivity, while genes within each module are functionally related; (ii) BeME-WithCo, which combines mutual exclusivity between modules with co-occurrence within modules; and (iii) BeCo-WithMEFun, which ensures co-occurrence between modules, while the within module relations combine mutual exclusivity and functional interactions. We formulated the BeWith framework using Integer Linear Programming (ILP), enabling us to find optimally scoring sets of modules. Our results demonstrate the utility of BeWith in providing novel information about mutational patterns, driver genes, and pathways. In particular, BeME-WithFun helped identify functionally coherent modules that might be relevant for cancer progression. In addition to finding previously well-known drivers, the identified modules pointed to other novel findings such as the interaction between NCOR2 and NCOA3 in breast cancer. Additionally, an application of the BeME-WithCo setting revealed that gene groups differ with respect to their vulnerability to different mutagenic processes, and helped us to uncover pairs of genes with potentially synergistic effects, including a potential synergy between mutations in TP53 and the metastasis related DCC gene. Overall, BeWith not only helped us uncover relations between potential driver genes and pathways, but also provided additional insights on patterns of the mutational landscape, going beyond cancer driving mutations. Implementation is available at https://www.ncbi.nlm.nih.gov/CBBresearch/Przytycka/software/bewith.html

## Author summary

The genomic landscape of cancer obtained from large scale studies revealed mutational patterns such as mutual exclusivity and co-occurrences. Despite significant efforts, the understanding of the patterns harbored by specific genes and their interplay with functional interactions are still limited. Both mutual exclusivity and co-occurrence can arise due to several reasons. For example, two functionally interacting genes may dysregulate the same cancer related pathway when either of them is mutated, leading to mutually exclusive mutations. Alternatively, mutual exclusivity might reflect mutations specific to two different cancer types. Methods for joint analysis of co-occurrence, mutual exclusivity, and functional interaction relationships can lead to a better understanding of the causes and impacts of mutations in cancer. We report a new computational approach, BeWith, which identifies groups of genes (or gene modules) with coherent patterns within modules, but distinct properties among genes in different modules. The general formulation of our method allows us to investigate various aspects of the cancer mutational landscape, leading to uncovering relationships between mutated gene modules, cancer subtypes, and mutational signatures.

“This is a *PLOS Computational Biology* Methods paper.”

## Introduction

The analysis of the mutational landscape of cancer has been instrumental in studying the disease and identifying its main drivers and subtypes. In particular, mutual exclusivity of mutations in cancer drivers has recently attracted a lot of attention. This relation can help identify cancer drivers, cancer-driving pathways, and cancer subtypes [1–10]. Although less studied, co-occurrence of mutations can also provide critical information about possible synergistic effects between pairs of genes [11–13] or underlying mutagenic processes [14–16].

Importantly, both properties can arise due to several different reasons, making the interpretation of the implied gene-gene relations challenging. Specifically, mutually exclusive mutations within functionally interacting genes may indicate that a mutation in either of the two genes dysregulates the same pathway. On the other hand, mutually exclusive mutations might also reflect a situation where mutations in two genes are associated with two different cancer types or subtypes. We have previously observed that within cancer type mutual exclusivity is more enriched with physically interacting pairs of genes compared to between cancer type mutual exclusivity [3]. Thus, the presence or absence of interactions between genes with mutually exclusive mutations might provide hints toward the nature of mutual exclusivity. In addition, the property of mutual exclusivity of mutations is not necessarily limited to cancer drivers, and therefore a proper understanding of this property is critical for obtaining a better picture of the cancer mutational landscape, both in general and for cancer driver prediction.

As with mutual exclusivity, co-occurrence of mutations might emerge due to a number of different causes. One of the most important cases is when simultaneously disabling two genes might be beneficial for cancer progression. Examples of such a scenario include the co-occurrence of TP53 mutation and Myc amplification [12,17] or co-occurring mutations in PIK3CA and RAS/KRAS [5,11,18]. An alternative explanation for the co-occurrence of somatic mutations might be the presence of a common mutagenic process. If patients were exposed to the same mutagen, the process might have left its footprint in regions susceptible to the mutagenic process. For example, we observed in the previous work that the co-occurrence of TP53 and TTN mutations in breast cancer patients was not statistically significant based on a test corrected with the patients’ mutation frequencies, although they were found to co-occur using an uncorrected Fisher’s exact test [16]. This suggests that the co-occurrence of mutations in TP53 and TTN are due to the fact that both genes were affected by a common mutagenic process acting on them, and not due to a benefit to cancer progression from mutations in both genes. Examples of mutagenic processes include the presence of APOBEC activities, aging, smoking, and deficiency of DNA damage repair process. Recent studies have found that many such processes are associated with different mutational signatures [14,15]. In addition, mutagenic processes can be context specific, leading to differences in mutational signatures even within the genome of an individual patient.

Given the diversity of reasons for observing the mutual exclusivity and co-occurrence relations, we hypothesised that jointly considering co-occurrence, mutual exclusivity, and functional interaction relationships will yield a better understanding of the mutational landscape of cancer. As a step in this direction, our goal was to develop a method to identify groups of genes (or gene modules) that show coherent patterns within modules, but distinct properties with genes outside modules. While many methods to identify cancer related modules exist, such modules are typically identified by focusing on the relationships of genes within a module. In particular, there have been several previous attempts to combine mutual exclusivity and functional interactions for module identification [1–3,19]. However, most of these methods were primarily focused on finding functional modules that include genes with mutually exclusive mutations without considering the relationships between such modules.

To address this challenge, we designed a general framework, named BeWith, for identifying modules with different combinations of mutation and interaction patterns. On a high level, BeWith tackles the following problem: given a set of genes and two types of edge scoring functions (within and between scores), find clusters of genes so that genes within a cluster maximize the “within” scores, while gene pairs spanning two different clusters maximize the “between” scores. We formulated the BeWith module identification problem as an Integer Linear Programming (ILP) problem and solved it to optimality. The flexibility of the ILP formulation allowed us to include additional constraints, such as module density, to enhance the module discovery process (See Methods for the description of ILP formulation).

**Fig 1:**
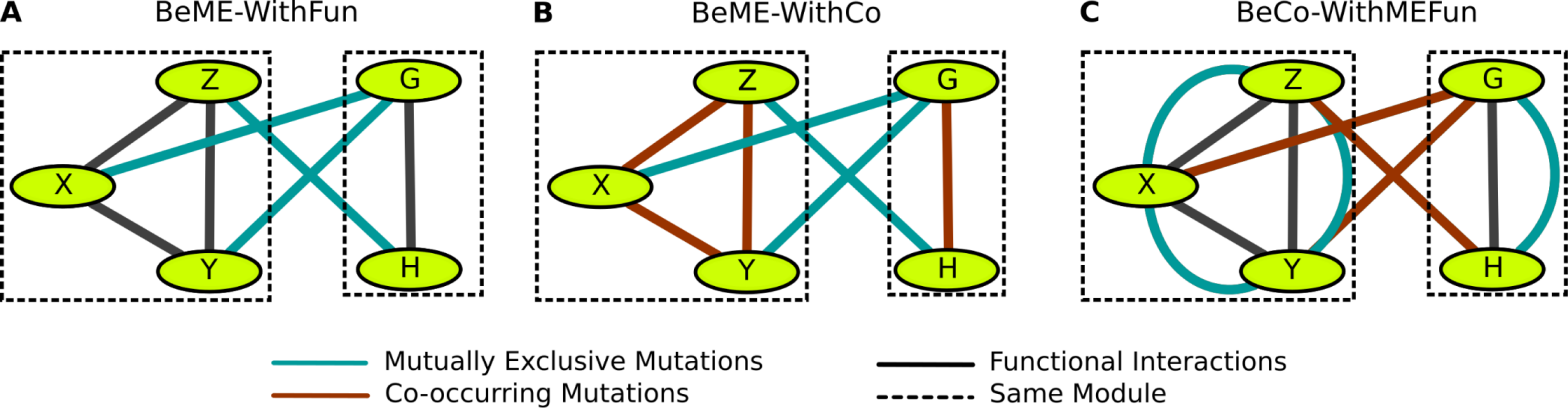
Overview of three settings for which BeWith was applied. (A) The goal of BeME-WithFun is to discover modules which have dense functional interactions within the modules while having mutually exclusive mutations with genes in other modules (B) In BeME-WithCo we aim to identify modules which have co-occurring mutations within the modules while having mutual exclusivity between the modules (C) In BeCo-WithMEFun, we look for modules of functional and mutual exclusivity relations inside a module with co-occurring mutations between modules.

As we consider different combinations of interactions for between and within scores, there are many possible settings to which we can apply the BeWith framework. In this work, we focused on the following three settings of the BeWith framework, in each of which we expect to uncover a set of modules with different biological properties (Fig 1). Below we provide a brief overview of the settings and defer the detailed explanation of each setting to Results Section.

Setting 1 (BeME-WithFun): Our first setting (Fig 1A) uses functional interactions to make sure genes in the same pathway are clustered together by rewarding within functional interactions and penalizing between functional interactions. At the same time, we reward mutual exclusivity without functional interactions between modules. Different from many previous attempts to utilize mutual exclusivity for the identification of functional modules dysregulated in cancer [1–10], here we ask the question of whether there exist functional modules that show mutual exclusivity between modules. Such modules, if found, could suggest subtype specific modules or be related to a mechanism similar to between pathways epistasis in a cancer setting [20].

Setting 2 (BeME-WithCo): This setting searches for groups of genes that are co-mutated (within), exclusively compared to other groups (Fig 1B). There are several possible explanations for co-occurring mutations. First, such co-mutations may be observed in cancer patients because of possible benefit to cancer progression. For example, deficiency in the DNA damage repair machinery by itself does not cause cancer, but rather makes a cell vulnerable to cancer causing mutations. Interestingly, if a co-mutation is indeed beneficial for cancer progression, then the mutations in turn might lead to a pattern of mutual exclusivity with other cancer driving mutations, as we look for in this setting. Second, co-mutation may arise because a mutational process affects a subset of genes disproportionately. Indeed, recent analysis has found that mutational signatures due to different mutagenic processes (such as aging, smoking, and APOBEC activity) can be observed in cancer patients [14]. In this context, ensuring mutual exclusivity between modules can help separate groups of genes affected by different mutagenic processes. In either case, combining co-occurrence within modules and mutual exclusivity between modules can lead to a more insightful understanding of the cancer mutational landscape. This setting is another novel way of analyzing the patterns of cancer mutations, using co-occurrence within modules.

Setting 3 (BeCo-WithMEFun): Our third setting is specifically designed to identify co-occurring driver pathways. We seek modules displaying functional and mutual exclusivity relations inside a module and co-occurrence between modules (Fig 1C). Leiserson *et al*. presented an anecdotal example of such co-occurring pair of modules (of two genes in each) in their GBM data analysis [5], which we were able to identify using this BeWith setting as discussed below.

We applied BeWith in the above three complementary settings to two TCGA datasets: somatic mutation profiles in breast cancer (BRCA) and endometrial cancer (UCEC).

## Results

### Method evaluation

To validate our method, we computed the significance of the results compared to those obtained with 100 randomized instances. To evaluate the effectiveness of using both between and within scores, we also used randomization of specific types of edges only (See Section C in S1 Text for additional details of the methods used to generate random instances). We evaluated the modules obtained in each setting for the objective function value and how well the modules identified known driver genes. Although BeWith is not specifically targeted toward detecting cancer driving genes, but rather searching for gene modules that may expose various biological properties, our modules are still expected to be enriched with cancer related genes. Indeed, we found that our modules significantly outperformed the random ones with respect to all measures (Table 1 for BRCA and Table 2 for UCEC). Randomizing only functional/co-occurring edges but keeping mutual exclusivity information has led to finding many singleton modules that are mutually exclusive with each other (essentially finding a set of genes with mutually exclusive mutations). Therefore for this setting, we performed the comparison using non-trivial modules (of size > 1) only. We did not include setting 3 in the evaluation because this setting only identified a small number of genes. For the list of cancer drivers, we used a combined list from COSMIC Cancer Gene Census [21] and 138 cancer driver genes from [22].

**Table 1:** Comparison of the results of three settings of the BeWith schema on real and randomized BRCA data. For the genes identified in each setting, we computed the number of known drivers, enrichment p-value, and objective function value. In addition, we provided the significance of each value by comparing it with those obtained with 100 randomized instances and computing empirical p-values.

	Features	# Known Drivers	Driver Enrichment (Hypergeometric test)	Objective Function Value
**BeME-WithFun**	**Real**	**14**	**6.9e-8**	**57.60**
	Randomization of all edges (average)	3.85 (*p* <0.01)	0.074 (*p* <0.01)	9.92 (*p* <0.01)
	Randomization of functional edges only	8.37 (p<0.01)	9.57e-4 (p = 0.03*)	31.49 (p<0.01)
	Randomization of ME edges only	6.31 (p<0.01)	0.03 (p<0.01)	20.17 (p<0.01)
**BeME-WithCo**	**Real**	**7**	**2.41e-3**	**43.79**
	Randomization of all edges (average)	2.55 (*p* <0.01)	0.17 (*p* = 0.02)	10.75 (*p* <0.01)
	Randomization of CO edges only	4.81 (p<0.01)	0.69 (p<0.01*)	19.35 (p<0.01)
	Randomization of ME edges only	3.20 (p = 0.01)	0.13 (p = 0.05)	14.93 (p<0.01)

* p-values computed for the subset of modules with more than one gene.

**Table 2:** Comparison of the results of the three settings of the BeWith schema on real and randomized UCEC data. We also performed additional evaluation of our method by running BeWith on simulated data to validate if BeWith indeed find modules with the desired properties in presence of random noise (See Section C in S1 Text for discussion). The results demonstrated that our method is robust and recover the planted modules in most instances (≥99%). The accuracy decreased if the planted module was increasingly noisy (See Table A in S1 Text). In addition, we compared our modules with the modules identified by other module detection algorithms and showed that our method is better than or comparable to previous methods in terms of cancer driver identification, while our modules have additional properties such as co-occurrences and functional coherence (See Section D in S1 Text).

	Features	# Known Drivers	Driver Enrichment (Hypergeometric test)	Objective Function Value
**BeME-WithFun**	**Real**	**10**	**1.53e-4**	**116.08**
	Randomization of all edges (average)	3.75 (*p* <0.01)	0.08 (*p* = 0.03)	15.17 (*p* <0.01)
	Randomization of functional edges only	1.8 (p<0.01)	0.36 (p = 0.02) (*)	15.09 (p<0.01)
	Randomization of ME edges only	4.70 (p<0.01)	0.05 (p = 0.05)	21.05 (p<0.01)
**BeME-WithCo**	**Real**	**7**	**6.05e-4**	**84.55**
	Randomization of all edges (average)	2.55 (*p* <0.01)	0.17 (*p* = 0.02)	10.75 (*p* <0.01)
	Randomization of CO edges only	3.85 (p<0.01)	0.38 (p = 0.03) (*)	27.49 (p<0.01)
	Randomization of ME edges (average)	1.79 (p<0.01)	0.24 (p<0.01)	15.09 (p<0.01)

### BeME-WithFun: Functional modules with mutual exclusivity between modules

#### Motivation

In this setting (Fig 1A), we search for functionally related groups of genes with potential relevance to cancer, using functional interactions and mutual exclusivity information. Our setting is different from most previous module detection methods based on mutual exclusivity in the sense that we do not assume that a group of genes with mutually exclusive mutations is necessarily in the same pathway. While pairs of genes with mutually exclusive mutations have been shown to be enriched among many cancer drivers, some of those genes are found to not be in a direct functional relation (such as prominent cancer drivers TP53 and GATA3). As we noted in the introduction, mutual exclusivity can arise due to various reasons other than dysregulating the same pathways. We hypothesize that mutually exclusive pairs are likely to be relevant to cancer progression whether or not their partners are in the same pathways, and asked whether mutual exclusivity observed between distantly functionally related genes leads to modules associated with cancer.

Starting with this question, we identify modules within which genes that are functionally related and at the same time between which genes that show mutual exclusivity. Note that while genes within a module may also be mutually exclusive with each other, we do not optimize for within module mutual exclusivity nor do we penalize it. Instead, we enforce functional edges within modules and penalize functional interactions of genes between different modules to ensure that genes within the same module are likely to be in the same pathway. We also reward the edges between modules for mutual exclusivity, with which we expect to uncover functional modules that are mutually exclusive with each other. Such modules could suggest subtype specific mutations or be related to a mechanism similar to between pathways epistasis in cancer.

#### Results

**Fig 2:**
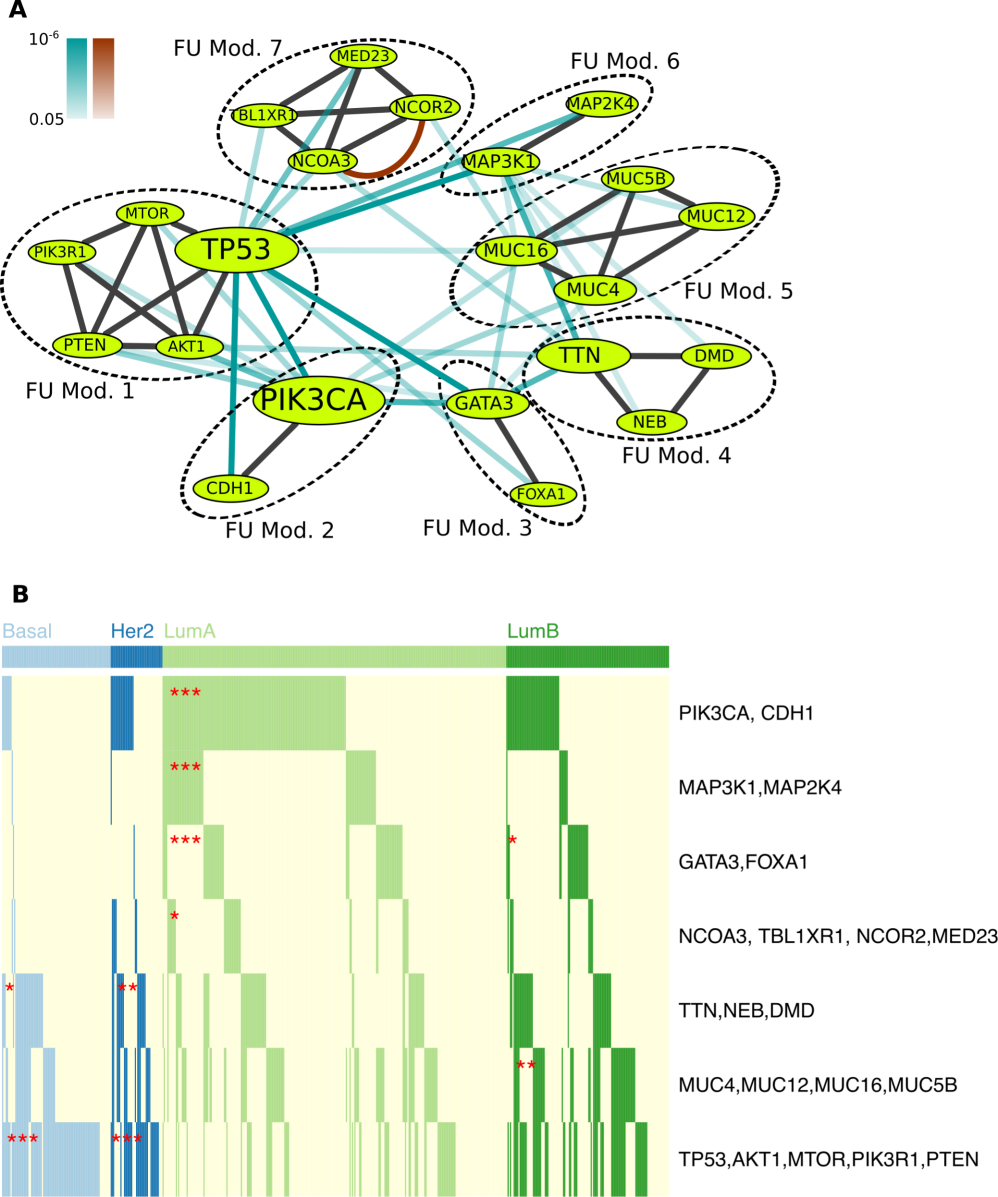
The Results in BeME-WithFun Setting for TCGA BRCA Dataset. (A) Modules uncovered by BeME-WithFun for TCGA breast cancer dataset. Cyan and brown edges represent pairs of genes with significantly mutually exclusive and co-occurring mutations, respectively. Darker edges correspond to lower p-values and p-values less than or equal to 1e-6 have same darkness. Black edges represent functional interactions. The node sizes of genes reflect the number of mutated samples. (B) The mutated samples for each subtype for the modules identified in BeME-WithFun. * indicates the significance of subtype enrichment relative to the overall mutations of a given module across all the subtypes (*** for p < 0.01, ** for p < 0.05, * for p < 0.1).

We applied this setting to two datasets: TCGA BRCA and UCEC somatic mutation dataset (See Methods for the description of the ILP formulation and definitions, and Section A in S1 Text for additional details of parameter selection). For the BRCA dataset, we identified seven modules (Fig 2A) including many known drivers in breast cancer, such as TP53, AKT1, CDH1, PIK3CA, GATA3, and MAP3K1 (in modules 1, 2, 3 and 6). Notably, many of the mutual exclusivity relations between modules we identified are among different pathways. As one exception, we note that despite being separated, modules 1 and 2 are closely related functionally since PIK3CA is a member of the PIK3CA/AKT1/MTOR pathway. Although they are functionally well connected, the algorithm split them into two groups as we maximized mutual exclusivity between modules, and the strong mutual exclusivity between modules outweighed the functional relationships.

The results reveal that mutual exclusivity, although commonly sought for within pathways, may also frequently occur between pathways. In addition, since BeME-WithFun strictly enforces functional interactions within the modules, we obtain functionally coherent modules. In particular, TP53 and GATA3 are typically put together into one mutual exclusivity module [4,5] even though they are only distantly functionally related. Overall, by the design of our method, the BeME-WithFun modules are densely connected (distance = 1.03) in terms of functional interactions compared with an average distance of 2.52 for Multi-Dendrix and 1.08 for MEMCover on BRCA mutation dataset. Benefiting from the combined analysis with functional modules, the method also allowed us to identify less frequently mutated drivers such as MED23, FOXA1, PIK3R1, and genes previously not implicated in breast cancer such as MTOR (we consider genes not reported in [23] as novel in breast cancer).

Interestingly, module 7 contains two genes with co-occurring mutations: NCOR2 (nuclear co-repressor) and NCOA3 (coactivator), with the latter being a well-known cancer driver [22]. However, neither of the two genes has been previously associated with breast cancer. TBLR1 is another nuclear co-repressor not previously reported to be associated with breast cancer, and MED23 is a component of the mediator complex and a coactivator involved in regulated transcription. The module was probably not detected by previous methods due to the co-occurrence between NCOR2 and NCOA3, as most previous methods enforce mutual exclusivity within modules.

Although not including known cancer drivers, module 5 contains a cluster of 4 Mucins—members of a family of large proteins which are components in most gel-like secretions, with some involved in signaling. Mucins have been typically associated with cancer via abnormal expression. For example, MUC4 is proposed to contribute to tumor progression by promoting cell survival [24–26]. As a cautionary note, TTN and Mucins are very long genes. Yet, the mutual exclusivity pattern of the genes is prominent even after considering the length of the genes, and our previous studies found that the increased mutations in TTN in breast cancer are possibly linked to APOBEC activity [16]. A network based analysis also provided supporting evidence of TTN mutations as a disease marker [27].

We next examined the association of the modules with traditional breast cancer subtypes [28] (Fig 2B and Table C in S1 Text) as well as their association with mutagenic processes independent of the traditional subtyping. We found that the mutations in the PIK3CA/CDH1, MAP3K1/MAP2K4, FOXA1/GATA3 module are significantly enriched in the Luminal A subtype (p < 0.0l, Fisher’s exact test). The NCOA3/TBL1XR1/NCOR2/ MED23 module is moderately associated with the Luminal A subtype (p < 0.1). It is important to note that the enrichment is relative to the overall mutations across all the subtypes for a given module. In addition, the enrichment of modules with low mutation rates (e.g., NCOA3 module) may be underestimated due to the lack of statistical power. The association of the GATA3/FOXA1 module with the Luminal subtype is consistent with the fact that they co-regulate the expression of genes essential for luminal mammary epithelial cell development [29–32]. Strong association of both MAP3K1 and MAP2K4 genes with the Luminal A subtype is in agreement with the previous finding that mutations in MAP2K4 produce perturbations similar to MAP3K1 loss [33]. Mutual exclusivity between mutations in these genes (Figure C in S1 Text) further supports this interpretation.

On the other hand, the mutations in the TTN/NEB/DMD module is enriched in the Her2 (p < 0.05) and Basal (p < 0.1) subtypes, and Mucins are mutated significantly in the Luminal B subtype (p < 0.05). An interesting property of the modules containing TTN and Mucins is an underrepresentation of mutations in the Luminal A subtype. (Note also that while both modules are mutually exclusive with several other modules, they are not mutually exclusive with each other, Figure C-A in S1 Text). As we have previously linked mutations in TTN to the APOBEC mutagenic process [16], the under-representation is consistent with the observation that APOBEC level is lower in the Luminal A subtype relative to its activity in other subtypes [34], and thus these modules might be related to replication stress [35]. Finally, the module including TP53 is strongly associated with the Basal and Her2 subtypes (p < 0.01) predominantly due to mutations in TP53.

We also examined whether the modules identified by BeME-WithFun are more significantly mutually exclusive with other genes when compared to the mutual exclusivity of the modules’ individual genes. The confidence of the mutual exclusivity test is largely limited by the number of mutated samples, causing the patterns in rarely mutated genes to be hard to observe. Merging genes in the same module into one supergene, we computed the significance of mutual exclusivity between supergenes and other individual genes, allowing us to identify many new mutually exclusive pairs. For example, module 2, which contains PIK3CA and CDH1, is mutually exclusive with several genes implicated in cancer, including MED23 and DCC, a gene implicated in colorectal cancer but novel in the context of breast cancer (For the newly created supergene, we define that the supergene has a mutation in a patient if there is any mutated gene in the module for the patient. WeSME p-values were computed for the supergene and all other genes. WeSME is a mutation frequency aware, sampling based test for mutual exclusivity [16]). The mutual exclusivity of these two genes with either PIK3CA or CDH1 was not statistically significant, but was statistically significant with the supergene corresponding to module 2. Interestingly, module 5 (with Mucins) is mutually exclusive with many known breast cancer drivers including PIK3CA, MAP3K1, and RUNX1. The list of all statistically significant module-gene pairs where the statistical significance of the mutual exclusivity of the module-gene pair is higher than the mutual exclusivity of the given gene with any gene in the module is provided in Section I in S1 Text.

**Fig 3:**
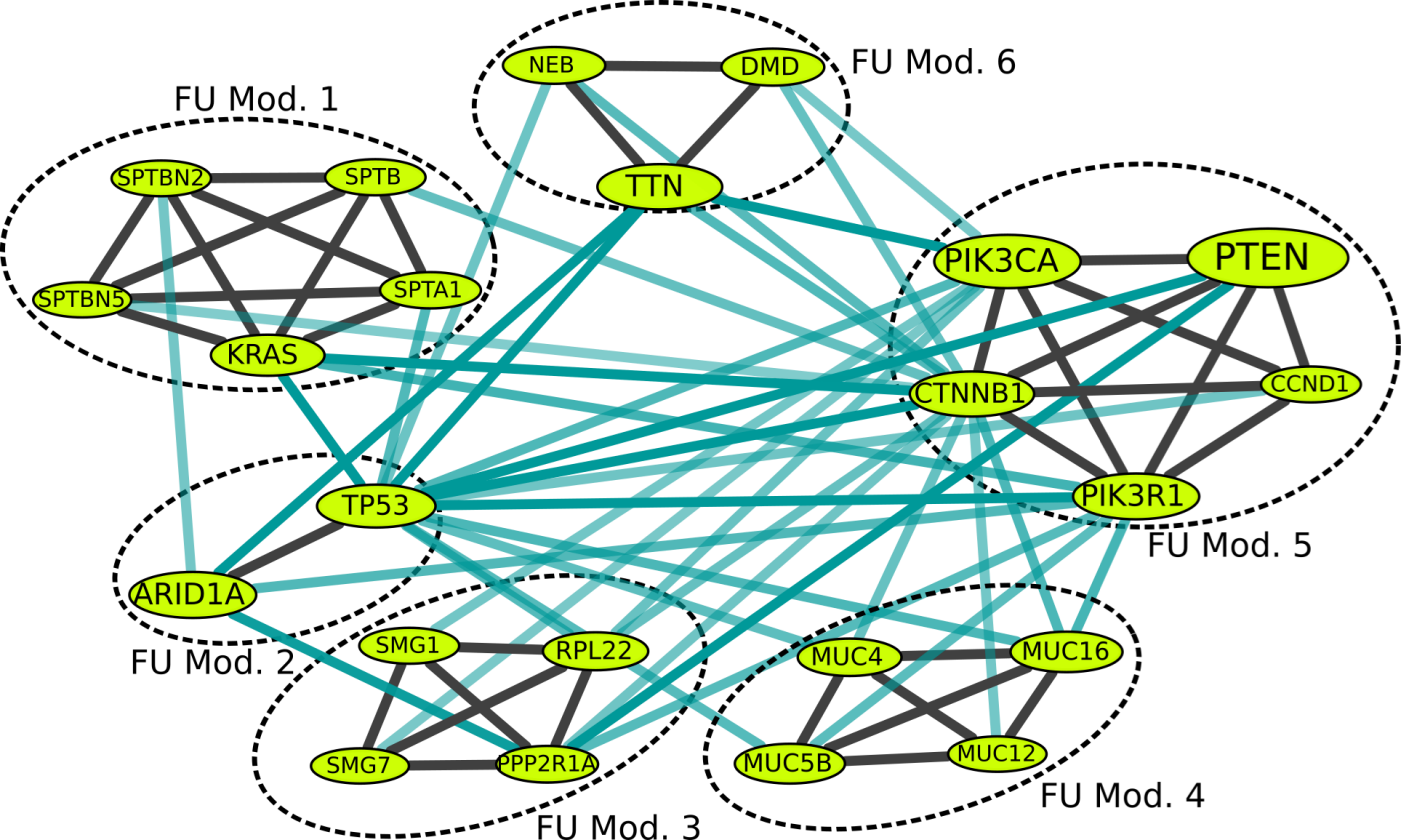
Modules uncovered by BeME-WithFun for Endometrial TCGA Dataset. Edge color-coding and node size coding are the same as in Fig 2A.

We also applied this setting to the TCGA UCEC somatic mutation dataset and identified six modules that include many prominent cancer drivers/associated genes in endometrial cancer such as TP53, CCND1, KRAS, PTEN, PIK3CA, PIK3R1, RPL22, and ARID1A (see Fig 3). BeME-WithFun also uncovered the same TTN and mucin modules as in the case of breast cancer. While it is possible that the increased number of mutations in these genes is related to their lengths, mutual exclusivity with respect to the mutations in other genes cannot be explained by gene lengths and requires an additional explanation.

As a novel finding, we also retrieved a module related to nonsense mediated decay (SMG1, SMG7). SMG7 is also known to regulate p53 stability and function in DNA damage stress response.

### BeME-WithCo: Co-occurrence modules that are mutually exclusive with each other

#### Motivation

We consider a second setting (Fig 1B), in which our goal is to find multiple co-occurrence modules. Two explanations have been proposed for the co-occurrence of mutations in cancer patients. First, such co-mutations of some genes might benefit cancer progression via their synergistic interactions. For example, deficiency in the DNA damage repair machinery by itself does not cause cancer but rather makes a cell vulnerable to cancer-causing mutations. Another possible explanation is that co-mutations arise when a mutational process causes mutations in some genomic regions more likely than in other regions. For the patients exposed to a particular mutagen, genes in the regions susceptible to the mutagenic process will be co-mutated. For example, mutations related to APOBEC activity would be enriched in genomic regions where the two DNA strands are more frequently separated (such as in replication origins, highly expressed genes, etc.). In contrast, mutations caused by the deamination of 5-methylcytosine would be more likely to occur in non-expressed genes since DNA methylation is associated with the silencing of gene expression [36,37]. Other examples of mutagenic processes include aging, smoking, and deficiency of the DNA damage process. Uncovering such mutational signatures can provide important insights on mutagenic processes affecting cancer patients [14,15]. Recently, several mutational signatures were identified in breast cancer patients, and a collection of identified signatures are available in Sanger COSMIC Signatures of Mutational Processes [23]. Below we show that some of modules we found indeed include unique mutational signatures.

Motivated by the fact that the co-occurrence of cancer mutations can be closely related to cancer progression, this setting searches for groups of genes that are co-mutated (within) and, at the same time, are mutually exclusive with genes in other groups (Fig 1B). While we are mostly interested in co-occurrence within modules in this setting, mutual exclusivity between modules helps separate groups of genes affected by different mutagenic processes. Combining co-occurrence within modules and mutual exclusivity between modules can lead to a more insightful understanding of the cancer mutational landscape. The setting is also very different from previous approaches of searching for genes with mutually exclusive mutations and is another novel way of analyzing the patterns of cancer mutations, using co-occurrence within modules.

#### Results

**Fig 4:**
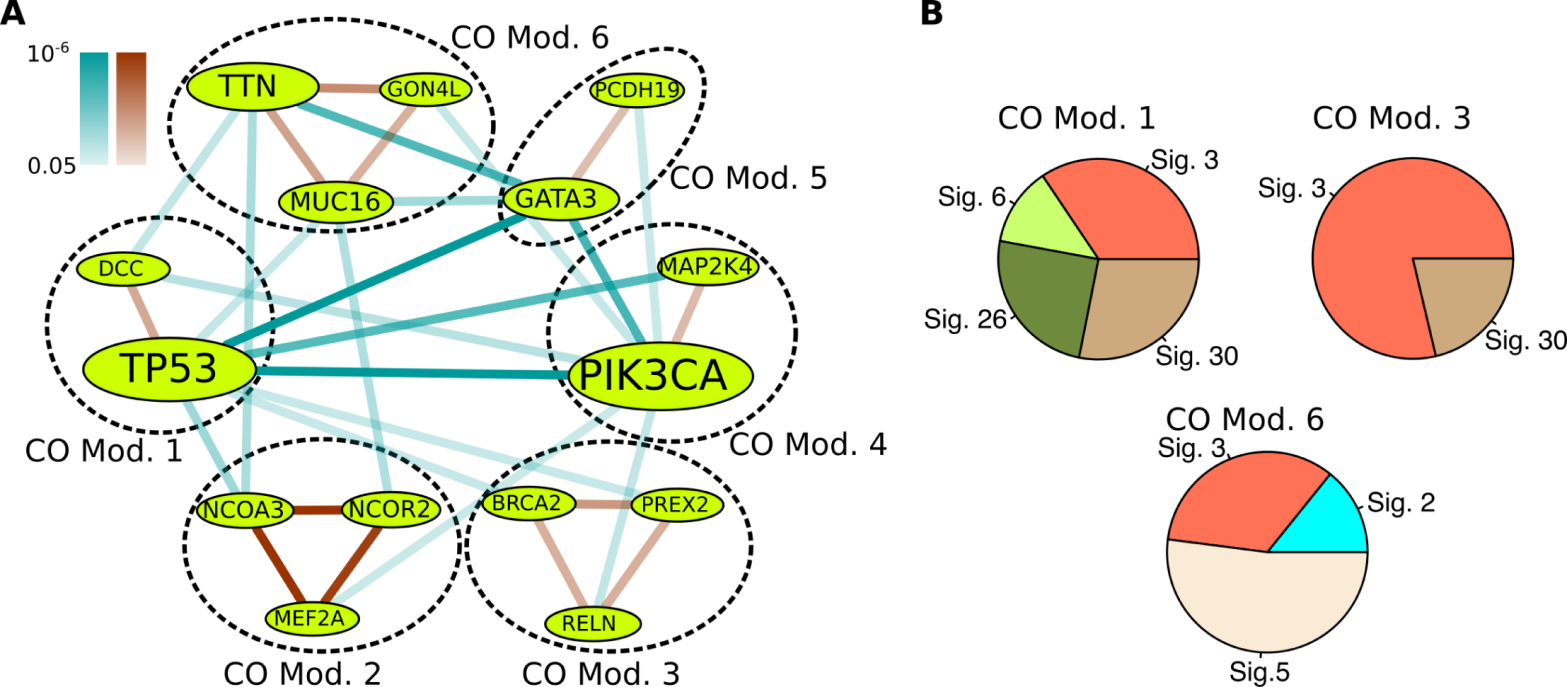
The Results in BeME-WithCo Setting for TCGA BRCA Dataset. (A) Modules uncovered by BeME-WithCo on breast cancer data. Edge color-coding and node size coding are the same as in Fig 2A. (B) Decomposition of the observed mutational spectra of modules 1, 3, and 6 into predefined COSMIC signatures of mutational processes identified in breast cancer. Signature 2 is APOBEC related, Signature 3 is associated with failure of DNA double-strand break-repair and also with BRCA1 and BRCA2 mutations. Signatures 6 and 26 are associated with defective mismatch repair. The aetiology of signatures 5 and 30 is unknown.

By applying BeME-WithCo to BRCA, we obtained six modules of genes (Fig 4A). As expected, the analysis of the modules in this setting revealed both types of co-occurring modules: modules containing putative cancer drivers with synergistic mutations and modules that are likely a result of common mutagenic processes. Interestingly, we found some modules with both properties, meaning that the genes in the module undergo similar mutational processes but their synergistic roles in cancer were also implicated in the literature.

Specifically, we found consistent mutational signatures in modules 1, 3, and 6 (Fig 4B, The remaining modules either did not have sufficient number of observed somatic mutations and/or their mutational spectrum could not be decomposed into signatures with a small error e.g. due to selection towards specific mutations.). Mutational signatures are distinctive patterns in a mutational spectrum that can reveal the underlying mutation generating processes [14,15]. See Section E in S1 Text for the method used to decompose mutational profiles into different signatures. It is interesting to see that different modules have different compositions of mutational signatures, which in turn implies that genes in different modules are affected by different mutagenic processes. The mutual exclusivity between modules in this setting facilitates the partitioning, if present in data. Uncovering such relations is important for a proper interpretation of mutual exclusivity, which can be extended to genes beyond cancer drivers. Note that in this setting we used the hypergeometric test for co-occurrence to allow the detection of modules due to the same mutagenic processes. We also applied the more stringent WeSCO test for the identified modules to test whether co-occurrence within modules is likely to be functional (see the detailed discussion for three representative modules presented below).

For module 1, the mutational signatures associated with DNA repair are the dominating signatures in this module (matched with Signatures 3, 6, and 26 from COSMIC Signatures of Mutational Processes in Human Cancer, http://cancer.sanger.ac.uk/cosmic/signatures). In addition, both genes in the module (TP53 and DCC) are known to be associated with cancer. TP53 is involved in DNA repair, growth arrest, and apoptosis. In particular, mutations in TP53 can lead to uncontrolled proliferation and invasive growth. On the other hand, DCC is suggested to have an anti-metastatic role [38], meaning that it may only contribute to cancer in the context of a preexisting condition. We conjecture that the mutations in DCC may be contributing to cancer progression for patients with defective mismatch repair and/or impaired TP53 functionality.

In contrast, module 3 is most strongly enriched with Signature 3, which is known to be associated with BRCA1 and BRCA2 germline and somatic mutations. The presence of BRCA2 in this module is consistent with the finding. Interestingly, the module includes PREX2, which has been recently identified as a negative regulator of PTEN in breast cancer [39]. In addition, the gene has been shown to be not only significantly mutated in human melanomas, but also relevant for melanoma tumorigenesis by a combination of mutations and overexpression [40]. However, the precise mechanism(s) of action remains unknown. The inclusion of PREX2 in the cluster with the BRCA1/2 mutation pattern might shed some light on possible synergistic interactions of this recently proposed driver.

Different from the above two modules, module 6 contains three long genes including MUC16 and TTN. An interesting aspect of this cluster is the presence of an APOBEC related signature (Signature 2), but no mismatch repair associated signatures. For more discussion of this and the remaining modules, see Section F in S1 Text.

### BeCo-WithMEFun: Mutually exclusive modules that are co-occurring with each other

Our third setting is specifically designed to identify co-occurring driver pathways by seeking modules displaying functional and mutual exclusivity relations inside a module and co-occurrence between modules. This setting is motivated by the fact that a single mutation may be enough to cause pathway dysregulation (thus mutual exclusivity within a module), and multiple dysregulated pathways are required for cancer progression. It is most similar to the traditional way of looking at mutually exclusive modules (Fig 1C). For example, Leiserson et al. presented an interesting example of such co-occurring pair of modules (CDK4/RB1 and TP53/MDM2) in GBM data analysis [5]. For validation, we applied BeWith to their GBM data (and the same criterion for co-occurrence/mutual exclusivity) in this setting and found that the setting successfully recovered the two modules.

Applying BeCo-WithMEFun to TCGA BRCA dataset, we identified a pair of modules: Module 1 with TP53 and BRCA2, and Module 2 with DCC (Figure E in S1 Text).

Both BRCA1 and BRCA2 are known to interact with TP53 and contribute to DNA repair and transcriptional regulation in response to DNA damage [41,42]. The activation of TP53 can also occur in response to DNA damage amongst other stresses. As discussed in the previous section, DCC is believed to have an anti-metastatic role, so its reduced functionality might have a synergistic effect with other cancer driving events. This observation is consistent with the finding in the BeME-WithCo setting, and points to a possible synergy between DCC and the broader DNA repair pathway. However, BeCo-WithMEFun did not find larger co-occurring modules in either of the two cancers types.

## Discussion

We introduced the BeWith framework to identify multiple mutated modules displaying specific mutation patterns between and within modules. In this work, we considered three settings: BeME-WithFun (ensuring mutual exclusivity of mutations between different modules and functional similarity of genes within modules), BeME-WithCo (ensuring mutual exclusivity between modules and co-occurrence of mutations in genes within modules), and BeCo-WithMEFun (ensuring co-occurrence between modules while enforcing mutual exclusivity and functional interactions within modules). By utilizing these different settings of within and between properties, BeWith revealed complex relations between mutual exclusivity, functional interactions, and co-occurrence. In particular, BeME-WithFun identified functionally coherent modules containing cancer associated genes, including previously unappreciated modules such as the NCOA3/NCOR2 module. Different from most of previous methods focusing on mutual exclusivity within modules, our first two settings enforce mutual exclusivity between modules. Interestingly, our modules still include many known cancer drivers (more than or comparable to previous methods), while they also exhibit significant mutual exclusivity relationships between modules. The BeME-WithCo setting also allowed us to investigate mutated modules in a novel way by looking for co-occurring mutations inside a module. This setting was particularly insightful in helping us uncover pairs of genes with likely synergetic effects in breast cancer. Going beyond cancer driving mutations, the setting provided additional insights into underlying mutagenic processes in cancer. Specifically, it revealed that different gene groups might differ with respect to their vulnerability to different mutagenic processes. The differences can contribute to strong mutual exclusivity signals between modules. Finally, while with BeCo-WithMEFun, we were able to elevate some of the observations obtained by BeME-WithCo to the pathway level, the setting did not uncover any larger co-occurring functional modules where the members of individual modules are mutually exclusive. The observation suggests that after conservative correction in the co-mutation test with mutation frequencies, co-occurrence of mutations in two different functional modules appears to be a rather rare event.

Overall, we demonstrated that BeWith can be used to uncover relationships between genes, gene groups, and pathways that were not accessible by previous methods. Importantly, the BeWith formulation is very general and can be used to interrogate other aspects of the mutational landscape by exploring different combinations of within-between definitions and constraints with simple modifications.

## Methods

We start by defining the Between-Within module finding (BeWith) problem, and then formulating it as an integer linear program. The optimization problem provides a general framework for identifying a set of clusters. By adjusting reward and penalty functions and some of the constraints, we can apply the framework to detect modules occurring in the different settings. In detail, we are given two weight functions between(*i*, *j*) and within(*i*, *j*) for pairs of genes between and within modules, respectively. We aim to identify a set of modules that maximizes between weights for gene pairs from different modules, while maximizing within weights inside a module simultaneously. The optimization problem is NP-hard (as it generalizes Max Cut), but can be solved optimally for current datasets, as we demonstrate below.

### General ILP formulation for the between-within module finding problem

Let *K* be the target number of modules, let *M* be the maximum number of genes per module, and let *V* be the set of genes we consider. We aim to group genes into one of *K* modules, where the (*K* + 1)-th cluster includes all unselected genes. Denote *K*′ = *K* + 1. We use the binary variable *y*_*ik*_ to indicate whether gene *i* is in module *k* (*y*_*ik*_ = 1) or *y*_*ik*_ = 0 otherwise. We define *ij* to be a between module pair if gene *i* and *j* are in two different modules *k*_1_ and *k*_2_, respectively (1 ≤ *k*_1_ ≠ *k*_2_ ≤ *K*) and to be a within module pair if both genes belong to the same module *k* (1 ≤ *k* ≤ *K*); *ij* is an unselected pair otherwise. Additionally, the following integer binary variables are used to capture different types of pairs:

- *x*_*ijk*_ = 1 if pairs *ij* is a within module pair and gene *i* and *j* are in the same module *k*, *0* otherwise.
- *z*_*ij*_ = 1 if pair *ij* is a between module pair, *0* otherwise.
- *u*_*ij*_ = 1 if pair *ij* is unselected, *0* otherwise.

The objective of ILP is defined as:

$$Max\;\sum_{ij}{between(i,j)\;z}_{ij}+\sum_{ij}\sum_{k=1}^{K}within(i,j)\;x_{ijk}$$ (1)

The constraints (2)–(3) ensure that each gene *i* belongs to exactly one of the modules and that the module size is bounded by *M*.

$$\sum_{k=1}^{K+1}y_{ik}=1\;\forall i\in V$$ (2)

$$\sum_{i\in V}y_{ik}\leq M\;\forall k\in [1,K]$$ (3)

The set of constraints (4)–(6) ensure that *x*_*ijk*_ = 1 if both *i*,*j* are selected to module *k*, 1 ≤ *k* ≤ *K*.

$$x_{ijk}\leq y_{ik}\;\forall ij,\forall k\in [1,K]$$ (4)

$$x_{ijk}\leq y_{jk}\;\forall ij,\forall k\in [1,K]$$ (5)

$$x_{ijk}\geq y_{ik}+y_{jk}-1\;\forall ij,\forall k\in [1,K]$$ (6)

Similarly, the constraints (7)–(10) ensure the proper assignment of *u*_*ij*_ and *z*_*ij*_.

$$u_{ij\;}\geq y_{iK\prime }\;\forall ij$$ (7)

$$u_{ij\;}\geq y_{jK\prime }\;\forall ij$$ (8)

$$u_{ij\;}\leq y_{iK\prime }+y_{jK\prime }\;\forall ij$$ (9)

$$z_{ij\;}=1-u_{ij}-\sum_{k=1}^{K}x_{ijk}\;\forall ij$$ (10)

In some settings, we also added additional constraints for ensuring that the density of the modules is at least *D*:

$$\sum_{j\in \{j\prime |\exists ij\prime \in E_{X}\}}y_{jk}\geq D(M-1)(y_{ik}-1)+D(\sum_{j\in V}y_{jk}-1)\;\forall i\in V,\forall k\in [1,K]$$ (11)

where *E*_*X*_ is a subset of gene pairs depending on the setting. The constraints ensure that each gene *i* in module *k* is connected with at least *D* fraction of genes in module *k* via edges in *E*_*X*_. Note that if *D* ≥ 0.5, the module is a connected subgraph since for any two non-adjacent vertices, they must have a common neighbor. We additionally required in some settings that for each gene *i* in a module, it has at least one edge in a certain type of subset of edges *E*_*Y*_ (e.g., mutual exclusivity or co-occurrence) with genes in other modules:

$$\sum_{j\in \{j\prime |ij\prime \in E_{Y}\}}z_{ij}\geq y_{ik}\;\forall i\in V,\forall k\in [1,K]$$ (12)

Finally, although all the variables *y*_*ik*_,*x*_*ijk*_,*u*_*ij*_,*z*_*ij*_ are required to be binary, it is sufficient to require the variables *y*_*ik*_ to be binary and leave the other variables *x*_*ijk*_,*u*_*ij*_,*z*_*ij*_ continuous in [*0*,*1*], which makes sure that all the variables in the optimal solution are binary but reduces the running time (See the proof in Section B in S1 Text). To improve the efficiency of the method, we implemented a symmetry breaking technique. Symmetry in ILPs not only allows for equivalent solutions but can also create multiple equivalent subproblems in branch-and-bound trees. These equivalent solutions and equivalent subproblems can lead to a significant increase in the running time and memory usage of branch-and-bound algorithms. We reduced the symmetry in solving our ILPs by adding constraints to restrict to a feasible solution set. For the details of our solution and its impact on the running time we refer to Section C in S1 Text.

### Application of BeWith to TCGA datasets

We applied BeWith in three complementary settings for two TCGA datasets: somatic mutation profiles in breast cancer (BRCA) and endometrial cancer (UCEC). With somatic mutation profiles of 665 BRCA samples and 207 UCEC samples (after removing ultra mutated samples), we first computed their mutual exclusivity and co-occurring relationships for each gene pair and constructed networks retaining only significant relationships. To this end, we used WeSME and WeSCO, which are efficient, weighted sampling based methods for testing mutually exclusivity and co-occurrence respectively, taking into account mutation frequencies of cancer samples [16]. Specifically, we constructed a mutual exclusivity network *G*_*ME*_ = (*V*,*E*_*ME*_), in which *E*_*ME*_ is a set of gene pairs that have significantly mutually exclusive mutations based on WeSME. A co-occurrence network *G*_*CO*_ = (*V*,*E*_*CO*_) was computed with hypergeometric tests or WeSCO tests, depending on the setting. In addition, we utilized functional interaction information in some settings, obtained from the STRING database [43].

The weights *w*_*F*_(*ij*), *w*_*ME*_(*ij*) and *w*_*CO*_(*ij*) for each pair are defined based on the protein functional interaction confidence scores and *p*-values from the mutual exclusivity and co-occurrence tests, respectively. The weights are set to be 0 if the edge does not exist. In the three BeWith settings we used different definitions of between and within functions, and slightly different variants of the constraints as described below.

### Settings for BeWith

#### Setting 1: BeME-WithFun

This setting searches for functionally related groups of genes with potential relevance for cancer. In order to ensure that genes within each module are likely to be in the same pathway, we enforce functional edges within modules while penalizing functional interactions of genes from different modules. We also reward mutual exclusivity between modules, so our optimization function is:

$$Max\;\sum_{ij}\sum_{k=1}^{K}{w_{F}(ij)\;x}_{ijk}+\sum_{ij}{(w_{ME}(ij)-w_{F}(ij))\;z}_{ij}$$

To strengthen the functional relationships among genes of the same module, we utilize the constraints (11) to ensure that each module is dense in the functional interaction network. In addition, we required that for each gene *i* in a module, it has at least some mutual exclusivity edge(s) with genes in other modules as in constraints (12) by setting *E*_*Y*_ = *E*_*ME*_.

$$\sum_{j\in \{j\prime |ij\prime \in E_{ME}\}}z_{ij}\geq y_{ik}\;\forall i\in V,\forall k\in [1,K]$$

### Setting 2: BeME-WithCo

In order to identify co-occurring modules, we perform BeWith enforcing the co-occurrence within a module but penalizing within module mutual exclusivity (*within*(*i*,*j*) = *w*_*CO*_(*ij*) − *w*_*ME*_(*ij*)). To capture co-occurrence modules that are biologically relevant, we reward mutual exclusivity relations between modules (*between*(*i*,*j*) = *w*_*ME*_(*ij*) − *w*_*CO*_(*ij*)). The objective function is then defined as follows:

$$Max\;\sum_{ij}{\;(w_{ME}(ij)-w_{CO}(ij))z}_{ij}+\sum_{ij}\;\sum_{k=1}^{K}(w_{CO}(ij)-w_{ME}(ij))\;x_{ijk}$$

To strengthen the co-occurrence within each module, we enforce that each module has dense co-occurring interactions by the constraints (11). Similarly to BeME-WithFun, we utilize the constraints (12) to enforce a stronger mutual exclusivity requirement (*E*_*Y*_ = *E*_*ME*_) among the modules.

#### Setting 3: BeCo-WithMEFun

Complementing the above analyses we utilized BeWith to look for modules that contain mutually exclusive and functionally related gene modules that might co-occur with other modules. Specifically, we enforce mutual exclusivity while penalizing co-occurring mutations within modules (*within*(*i*,*j*) = *w*_*ME*_(*ij*) − *w*_*CO*_(*ij*) + *w*_*F*_(*ij*)). Genes in different modules are rewarded for co-occurrence (*between*(*i*,*j*) = *w*_*CO*_(*ij*) – *w*_*ME*_(*ij*) – *w*_*F*_(*ij*)). The objective function is then defined as:

$$Max\sum_{ij}{(w_{CO}(ij)-w_{ME}(ij)-w_{F}(ij))z}_{ij}+\sum_{ij}\sum_{k=1}^{K}(w_{ME}(ij)-w_{CO}(ij)+w_{F}(ij))\;x_{ijk}$$

In order to ensure that genes within modules are likely to be in the same pathways, we ensure that each module is a dense subnetwork in the STRING functional interaction network using the constraints (11).

To strengthen the co-occurrence between modules and mutually exclusivity within each module, we additionally required that for each gene *i* in a module, it has at least some co-occurrence edge(s) with genes in other modules (*E*_*Y*_ = *E*_*CO*_):

$$\sum_{j\in \{j\prime |ij\prime \in E_{CO}\}}z_{ij}\geq y_{ik}\;\forall i\in V,\forall k\in [1,K]$$

## Supporting information

S1 Text Supplementary materials. Additional results and method description.
(PDF)
