# Supplementary material for: BeWith: A Between-Within method to discover relationships between cancer modules via integrated analysis of mutual exclusivity, co-occurrence and functional interactions
Source: PLoS Comput Biol. 2017 Oct 12;13(10):e1005695. doi: 10.1371/journal.pcbi.1005695 (PMC5638227; doi:10.1371/journal.pcbi.1005695)
Supplement: S1 Text — Additional results and method description. (PDF) [file pcbi.1005695.s001.pdf]

S1. Supplementary Material

A. Data and Parameter Selection

Processing mutation data: We utilized TCGA BRCA and UCEC somatic mutation datasets. After removing ultra mutated samples (> 1000 combined alterations), we obtained 665 BRCA samples and 207 UCEC samples. For both datasets, we performed WeSME tests for all gene pairs to estimate the significance of mutual exclusivity. WeSME is an efficient, mutation frequency aware testing based on weighted sampling for mutual exclusivity [1]. For co-occurrence, we performed both WeSCO and hypergeometric tests depending on settings. Similar to WeSME, WeSCO is a mutation frequency aware test for co-occurrence. It is more stringent than hypergeometric test and therefore can potentially remove some mutational patterns by chance. We used hypergeometric test for BeME-WithCo in which we want to identify mutational signature and performed WeSCO analysis for the identified modules. For both datasets, we consider genes mutated in at least 7 samples.
For the BRCA dataset, we retained 174 pairs of genes with $p \leq 0.05$ from WeSME in all settings. In BeME-WithCo, 1,891 co-occurrent pairs of genes ($p \leq 0.01$, hypergeometric test) were used for further analysis. In BeCo-WithMEFun, we retained 1,235 pairs of genes with WeSCO ($p \leq 0.05$). For the UCEC dataset, we utilized 280 pairs of genes with $p \leq 0.001$ from WeSME and 1,028 CO pairs of genes with $p \leq 0.001$ from hypergeometric tests. To run BeCo-WithMEFun on UCEC data, we retain 992 pairs of genes with WeSCO $p \leq 0.05$. For each significant pairs, we define the weight of the edge to be $min(-log_{100} p, 3)$ as the p-values of WeSME and WeSCO has the precision up to 10^{-6}. This sets the weights of edges at least 1 and gradually increased when the p-value gets smaller. The log base 100 was used to make sure that each gene pair with significant p-value less than 0.01 from statistical tests from mutation data has a heavier weight than STRING functional edges.

Functional interactions: To obtain the edge weights for functional interactions, we downloaded functional protein interactions from STRING database version 10.0. We used the interactions with high confidence scores (>=900), then divided by the maximum (1000) to obtain the functional interaction edge weight.

The number of clusters: Let $f(k)$ be the value of the objective function by setting the number of modules to k. We define $f(k)/k$ as the average module benefit. Starting with $k = 2$ and iteratively increasing by 1, we observe that $f(k)/k$ gradually increases but peaks around 4 or 5 then start decreasing. We either pick K or K+1 where $f(k)/k$ is maximized at K. The number of clusters is set to be K+1 when $f(k)/k$ is maximized at K and at $K + 1$ we do not observe a significant drop ($f(K + 1)/(K + 1) \geq (0.9 * f(K))/K$). Otherwise we pick K to be the number of modules in the final solution. Figure A in S1 Text shows that we stops at $k = 7$ for BeME-WithFun, $k = 6$ for BeME-WithCo on BRCA dataset, $k = 6$ for BeME-WithFun and $k = 5$ BeME-WithCo on UCEC dataset. We used $k = 2$ for BeCo-WithMEFun with both TCGA BRCA and UCEC datasets.

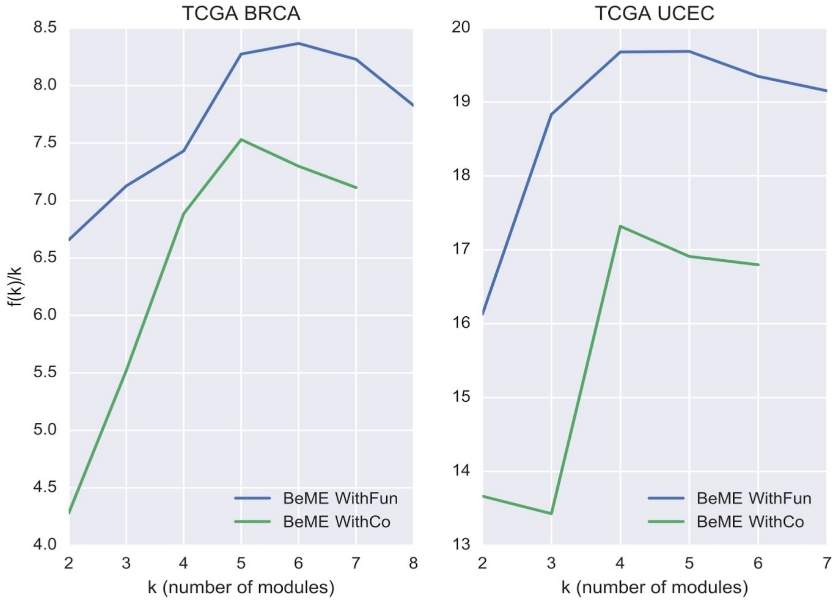

Fig. A The number of modules k versus average benefit per module $f(k)/k$ for TCGA BRCA and UCEC datasets.

<u>Density and maximum number of genes per module:</u> We set the density of the modules $D = 0.7$ to make sure that the majority of genes in the same module are functionally interacting or co-occurring. For the maximum number of genes per module , we set $M = 10$. Note that the number of genes in all of our modules on both BRCA and UCEC datasets is less than or equal to 5.

B. The Proof of Binary Constraints and Symmetry Breaking for Solving ILP

Binary Constraints

The binary constraints on y_{ik} is enough to make sure all the variables are binary in the optimal solution. Constraints (4) to (6) guarantee that x_{ijk} is binary if y_{ik} and y_{jk} are binary. This is because if either $y_{ik} = 0$ or $y_{jk} = 0$ then $x_{ijk} = 0$ by Constraints (4) and (5). When $y_{ik} = y_{jk} = 1$ then $x_{ijk} = 1$ by Constraints (6). Similarly, Constraints (7) to (9) guarantee that u_{ij} is also binary if $y_{iK'}$ and $y_{jK'}$ $(K' = K + 1)$ are binary. The set of constraints (10) guarantees z_{ij} is also binary given the u_{ij} and x_{ijk} are all binary.

Symmetry Breaking

Symmetry in ILPs can lead to significant increase in the running time and memory usage of branch-and-bound algorithms because it not only allows for equivalent solutions but can create multiple equivalent subproblems in branch-and-bound trees. Previous research have suggested adding constraints to restrict to feasible solution set in order to reduce the symmetry in solving ILPs [2,3].

A feasible solution to our ILP contains the assignments of the variables y_{ik} which assign gene i to module k. These assignments correspond to a 0-1 matrix where rows represent genes and columns represent modules. For example, Y_1 and Y_2 represent two feasible solutions when assigning 4 genes to 3 modules. However, Y_1 and Y_2 have the same objective value since we can permute the columns of Y_1 to obtain Y_2.

$$Y_1 = \begin{bmatrix} 1 & 0 & 0 \\ 0 & 0 & 1 \\ 0 & 0 & 1 \\ 0 & 1 & 0 \end{bmatrix}, \quad Y_2 = \begin{bmatrix} 1 & 0 & 0 \\ 0 & 1 & 0 \\ 0 & 1 & 0 \\ 0 & 0 & 1 \end{bmatrix}$$

To reduce the number of equivalent solutions, we restrict our feasible solutions to assignment matrices with columns in increasing lexicographical order. The column/module M_1 is lexicographically smaller than column M_2 if the smallest index of the genes of M_1 is less than that of M_2. In the above example, Y_2 has columns in increasing lexicographical order while Y_1 does not. In Y_1, column 3 is lexicographically smaller than column 2.

We can add the following constraints to the ILP to restrict the feasible region to assignment matrices with columns in increasing lexicographical order:

$$\sum_{l=k}^{K} y_{il} \leq \sum_{j=1}^{i-1} y_{j(k-1)} \qquad \forall i \in V \; \forall k \in [2, K], \; i \geq k$$

The above constraints ensure that we only assign gene i to one of the modules $k, k+1, ..., K$ if we already assign one of genes with smaller index to module $k-1$. Similar constraint sets were proposed to allocate surgery blocks to operating rooms [4].

As shown in Figure B in S1 Text, the running time of BeWith is significantly improved when adding symmetry breaking constraints for larger k. The running time of BeCo-WithMEFun is less than 10 seconds on both BRCA and UCEC datasets.

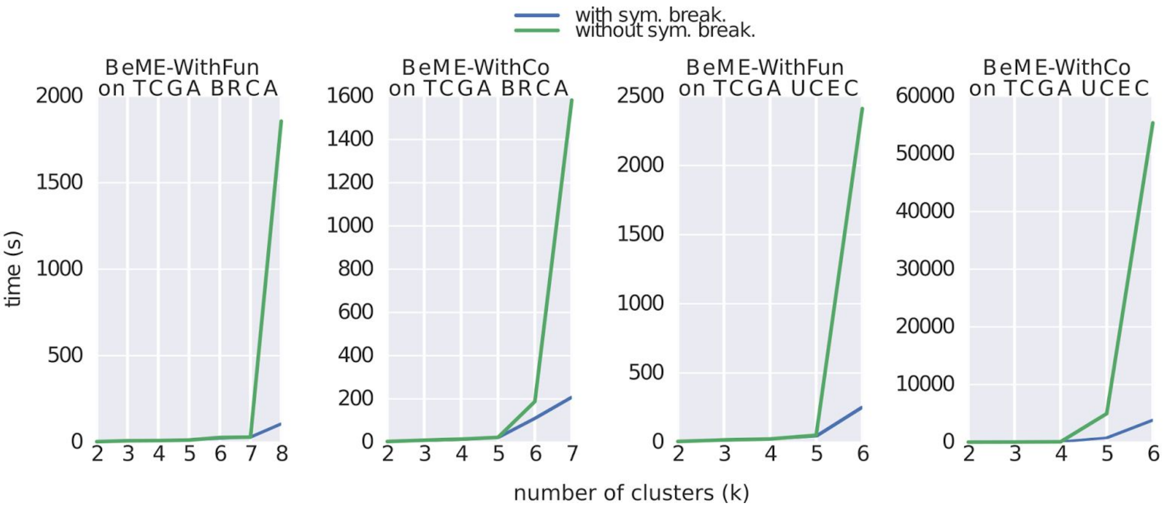

Fig. B. The running time of BeWith with and without symmetry breaking of BeME-WithFun and BeME-WithCo on TCGA BRCA and UCEC datasets.

C Method Evaluation

Generating random instances

For each randomized instance, we first randomize the STRING functional network by swapping edges, which preserves node degree distribution. For edge swapping, we randomly chose two edges without common neighbors and swap their neighbors so that two new edges are created and repeated the edge swapping process at least 100 times the number of edges as suggested by Milo *et al. [5]*. To randomize ME/CO edges, we permuted the p-values for the gene pairs with WeSME/WeSCO p-values less than 0.25. 100 random instances were generated to compute the significance of BeWith modules. When we do within or between only randomization, we randomized only some type of edges. For BeME-WithFun, we randomized only functional edges for within randomization and ME edge for between randomization. Similarly, for BeME-WithCo, we permuted only CO edge for within randomization and ME edges for between randomization

Generating Simulated inputs

To show how well BeWith can identify modules with desired properties in the presence of noise, we generated simulated data and applied BeWith to the generated data.

Step 1: Generating random background: We first created a randomized functional network of 1000 genes in this step as follows: Choose 1000 genes randomly from Human String network. Randomize the subnetwork of these genes by swapping edges. ME and/or CO edges among 1000 genes are then randomly added depending on the settings. Let P_{ME} and P_{CO} be the list of p-values from WeSME and WeSCO tests on TCGA Breast Cancer dataset. Consider each gene pair and randomly sample a p-value from the list of P_{ME} and add an ME edge if $p \leq 0.01$. CO edges are added similarly if they are used in the setting.

Step 2: Planting modules: We plant k $(2 \leq k \leq 4)$ subnetworks randomly in the random network generated in the previous step. We randomly choose k between 2 and 4 and for each module, we randomly choose 2-5 genes such that no genes belong to more than one module. Edges are added for the selected modules depending on the setting as follows.

BeME-WithFun: For each gene *g* in a module *M*, we choose a random fraction (at least 0.7) of other member genes in *M* and add functional edges among *g* and these genes. For each gene *g* in a module *M,* we randomly choose a random fraction of genes in other modules (at least one gene) and we add ME edges with significant p-values randomly sampled from P^S_{ME} where P^S_{ME} is the list of p-values (<=0.01) from WeSME on TCGA Breast Cancer dataset. When we add an ME edge between a pair of genes, we remove existing ME edge if there exists. Finally, with a probability r_n , we added random noise to each of planted significant edges by replacing the p-value of the ME edge with a random

p-value sampled from P^B_{ME} (the background p-values (>0.01) from WeSME on TCGA Breast Cancer dataset). We used varied noise levels r_n of 0, 0.1, 0.2 and 0.4 to evaluate how the accuracy changes depending on the noise level.

BeME-WithCo: For each gene *g* in a module *M*, we add CO edges among g and other genes in M . Each CO edge has a p-value sampled from P^S_{CO} where P^S_{CO} is the list of p-values (<=0.01) from WeSCO on TCGA Breast Cancer dataset. For each gene *g* in a module we randomly choose a random fraction of genes in other module (but at least one) and we add ME edges with p-values randomly sampled from P^S_{ME} . When we add an ME/CO edge between a pair of genes , we remove existing CO/ME edge if there exists. As with BeMEWithFun setting, we added noise to each planted CO/ME edge, with a given probability r_n by replacing its p-value with a random p-value sampled from the background p-values P^B_{CO} / P^B_{ME} .

BeCo-WithMEFun - For each gene *g* in a module *M*, we choose a random fraction (at least 0.7) of other member genes in *M* and add functional edges among *g* and these genes. Similarly, for each gene *g* in a module *M* we randomly choose a fraction of other member genes (at least one gene) in *M*, and add ME edges with p-values randomly sampled from P^S_{ME} . For each gene *g* in a module we randomly choose a fraction of genes in other module (at least one) and we add CO edges with p-values randomly sampled from P^S_{CO} . When we add an ME/CO edge between a pair of genes, we remove existing CO/ME edge if there exists. We added random noise to the planted edges similarly as in other settings.

We ran BeWith for each setting with stopping criteria as described in Section S1 and setting the within density $D = 0.6$ considering the noise in the simulation experiments. We use different noise levels r_n (0.1, 0.2 and 0.3) and evaluated the accuracy of our methods. For each noise level, we report the accuracy of our method (the fraction of instances we correctly retrieve the planted set of modules among 100 simulated datasets). In the case that we did not correctly retrieve the set of planted modules, we checked whether we can identify a partial solution that is the majority of the genes in the planted set of modules. We define our solution as partial match if our algorithm retrieved at least 75% of genes in the planted modules. We compute the partial match percentage as the ratio between the number of times we achieve partial match (including cases we achieve perfect match) and the number of times we do the experiments. We report the accuracy and the partial match percentage of our method in Table A.

Noise Level (r_n)	Measurements	BeME-WithFun	BeME-WithCo	BeCo-WithMEFun
0.0	Accuracy	0.99	1.0	0.99
	Partial Match Percentage	1.0	1.0	1.0
0.1	Accuracy	0.98	0.95	0.93

	Partial Match Percentage	1.0	1.0	1.0
0.2	Accuracy	0.95	0.78	0.84
	Partial Match Percentage	1.0	1.0	0.99
0.3	Accuracy	0.89	0.65	0.77
	Partial Match Percentage	1.0	1.0	0.98

Table A. The robustness results of BeWith with simulated data

As expected the accuracy of our method is inversely proportional to the level of the planted noise. In the cases we could not retrieve exactly the set of planted modules, BeWith is likely able to retrieve the majority ($\geq 75\%$) of genes in these planted modules.

D. Comparison with methods for cancer module discovery

Our approach is different from most of previous methods for mutated module identification as we focus on finding modules with relations both within and between modules. Given differences in the objectives, we performed the comparison for the purpose of establishing whether modules identified with *BeME-WithFun* have similar enrichment in cancer genes relative to the modules uncovered by other methods despite the fact that modules uncovered with *BeME-WithFun* are optimised with respect to different set of relations.

The most comparable approaches are Multi-Dendrix [6], MEMCover [7] and CoMDP [8]. These algorithms seek to find multiple functional modules based on mutational patterns, enforcing mutual exclusivity relation within modules. Multi-Dendrix and MEMCover identify such multiple modules assuming mutations may potentially co-occur between modules but without enforcing it [6,7]. CoMDP [8] attempted to ensure co-occurrences between the modules. Table B-A in S1 Text shows the comparison of our results in *BeME-WithFun* setting with the modules obtained from Multi-Dendrix and MEMCover using BRCA somatic mutation dataset. Multi-Dendrix looks for multiple modules by optimizing mutation coverage and mutual exclusivity, by which they implicitly aim to ensure functional similarity within the modules. MEMCover optimizes mutation coverage while utilizing functional interactions and mutual exclusivity within modules. Unlike the previous methods, *BeME-WithFun* insists on mutual exclusivity *between* modules while using functional interactions within modules. We set Multi-Dendrix to produce the same number of modules as *BeME-WithFun* and the core modules (combining the results with the maximum module size varied from 2 to 5) were used for comparison. For MEMCover, we obtained modules by setting each patient to be covered at least by 5 mutated genes and then chose the same number of modules as *BeME-WithFun* based on the best coverage. Table B-A in S1 Text shows that *BeME-WithFun* finds better or comparable modules in terms of cancer driver enrichment. As we explicitly enforce functional interactions within modules, the *BeME-WithFun* modules are more functionally coherent as expected. We also compared our results with C3. Due to the lack of some main functionality, we could not run the program with our data but instead we compared with the best module shown for the breast cancer dataset in the paper. There are 5 cancer genes (out of 20) for two C3 modules (p=0.1)

Features	#Known Drivers	Driver Enrichment (Hypergeometric test)	Functional Coherence (Distance)
**BeME-WithFun**	14	6.9e-8	1.03
**Multi-Dendrix**	9	1.1e-3	2.52
**MemCover**	13	3.63e-7	1.08

Table B-A. Comparison of module properties obtained with *BeME-WithFun,* Multi-Dendrix and MEMCover on breast cancer data.

We compared the UCEC results with other related methods in the Table B-B in S1 Text.

Features	# Known Drivers	Driver Enrichment (Hypergeometric test)	Functional Coherence (Distance)
**BeME-WithFun**	10	5.1e-4	1.0
**Multi-Dendrix**	10	5.31e-6	2.31
**MEMCover**	13	6.25e-8	1.76

Table B-B. Comparison of module properties obtained with *BeME-WithFun,* Multi-Dendrix and MEMCover usig UCEC dataset.

CoMDP considers the setting similar to BeCo-WithMEFun but their original results with CNV data identified co-occurring genes that can be attributed to insertion/deletion events in the same locus rather than to co-occurring pathways. With BRCA somatic mutation data and requiring the same number of genes as returned by our algorithm, CoMDP produced two modules: (TP53) and (TTN, USH2A). These included only one known cancer driver (TP53, p=0.33) compared to two drivers (p=0.056) obtained by BeCo-WithMEFun. Importantly, neither TTN nor USH2A significantly co-occur with TP53 after correcting for patient mutation frequencies, making these modules hard to interpret.

In summary, although BeWith is designed to identify gene modules with specific mutation patterns in cancer rather than to find cancer driving genes, the comparison with module finding approaches revealed that BeWith performed well in finding driver genes too.

E. Decomposition of Module Mutational Spectrum into Mutational Signatures

We collected all observed single-nucleotide variants in TCGA together with their immediate sequence context for all genes in each module. Mutational spectrum for each module was calculated. We decomposed the mutational spectra into predefined mutational signatures using the R package *deconstructSigs* (version: 1.8.0) [9]. Sanger COSMIC Signatures of Mutational Processes identified in breast cancer [10] were used for decomposition. The input exome data were normalized to the whole genome. Signatures were not extracted for Modules 2, 4, and 5 due to either small number of somatic

variants in module genes or large number of presumably selective mutations that lead to increased error in decomposition.

F. Application of BeWith to TCGA BRCA datasets

Additional information about BeME-WithFun modules

This section supplements the discussion of modules identified in BeME-WithFun Setting. In Table C in S1 Text we show the number of mutated samples for each module for each subtype. Figure C in S1 Text shows the distribution for each gene.

	**All**	**Basal (90)**	**Her2 (43)**	**LumA (285)**	**LumB (135)**
PIK3CA, CDH1	223<br>(205, 34)	8<br>(8, 0)	19<br>(19, 1)	152***<br>(136***, 30***)	44<br>(42, 3)
MAP3K1,<br>MAP2K4	69<br>(43, 27)	1<br>(1, 0)	1<br>(0, 1)	59***<br>(38***, 22 ***)	8<br>(4, 4)
GATA3, FOXA1	70<br>(60, 11)	1<br>(0, 1)	1<br>(1, 0)	46***<br>(40***, 7)	22*<br>(19, 3)
NCOA3,TBL1XR1,<br>NCOR2,MED23	48<br>(16, 8, 21, 11)	2<br>(2, 0, 2, 0)	6<br>(1, 1, 3, 1)	30*<br>(10, 6, 10, 8)	10<br>(3, 1, 6, 2)
TTN,NEB,DMD	126<br>(92, 21, 21)	27*<br>(22**, 4, 1)	15**<br>(11*, 2, 2)	50<br>(35, 11, 9)	34<br>(24, 2, 9**)
MUC4,MUC12,<br>MUC16,MUC5B	158<br>(59, 38, 55, 28)	26<br>(10, 9, 6, 6)	16<br>(8*, 2, 6, 1)	69<br>(25, 18, 25, 11)	47**<br>(16, 9, 18*, 10)
TP53,AKT1,<br>MTOR,PIK3R1,<br>PTEN	218<br>(180, 13, 13, 13,<br>20)	77***<br>(76***, 0, 1, 2, 5)	28***<br>(26***, 1, 2, 2, 1)	54<br>(27, 10*, 8, 6, 8)	59<br>(51*, 2, 2, 3, 6)

Table C. Number of mutated samples for each subtype for each module. The total number of mutated samples for 4 subtypes are shown in the first row. For each module, we computed the number of samples with mutations in at least one gene in the module (followed by the number of mutated samples for each gene in the module). * indicates the significance of subtype enrichment relative to the overall mutations of a given module (or a gene) across all the subtypes. (*** for $p < 0.01$, ** for $p < 0.05$, and * for $p < 0.1$)

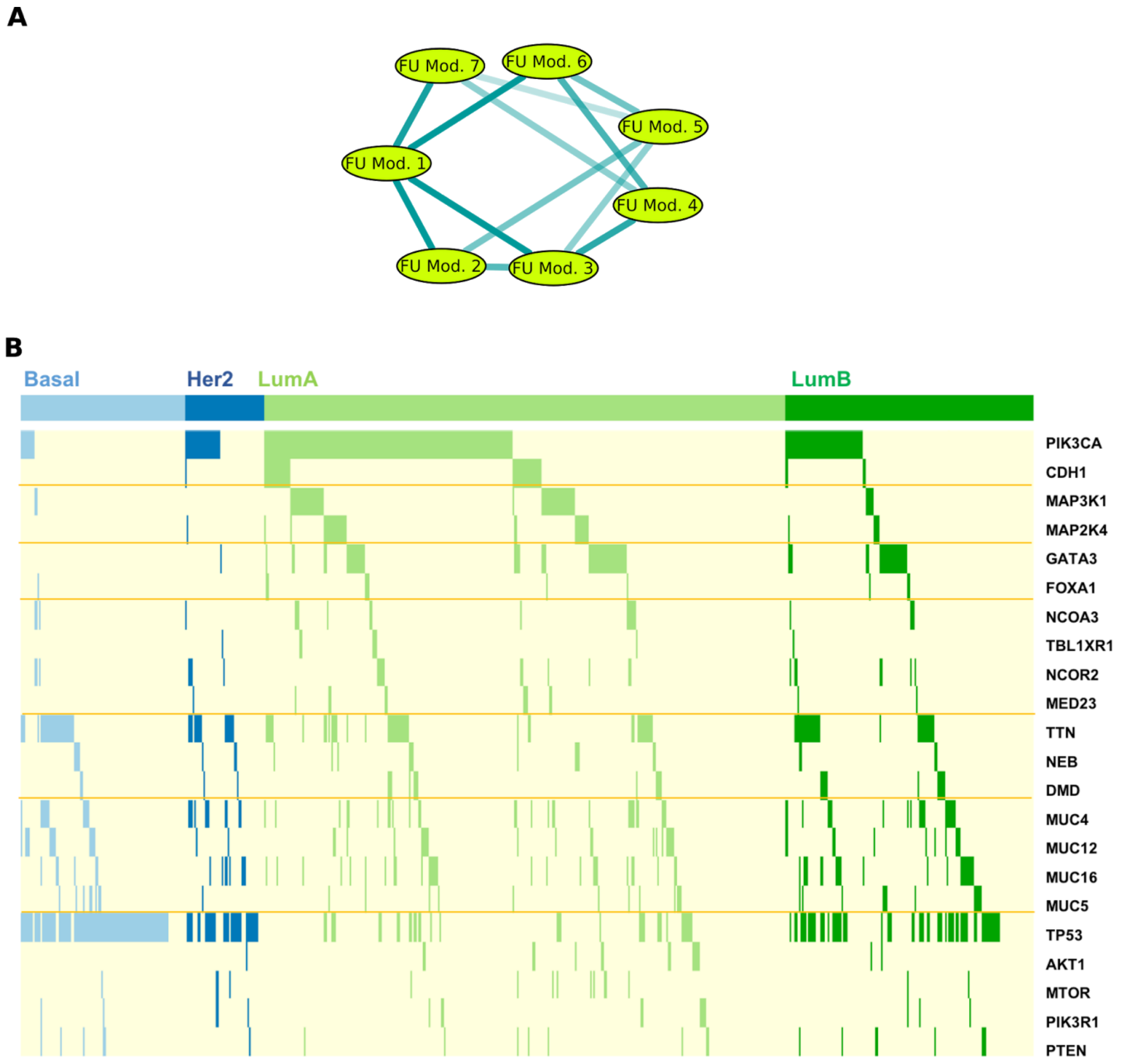

Fig. C. **(A)** WeSME p-values between the identified modules. There are many significant mutual exclusivity edges between modules and for some, the significance of mutual exclusivity is increased compared to the ones with gene pairs. (**B**) Mutated BRCA samples for each gene identified by BeME-WithFun.

Additional information about BeME-WithCo modules

This section supplements the discussion of modules in BeME-WithCo: co-occurrence modules that are mutually exclusive with each other in the main text (Figure C in S1 Text).

Module 2 includes NCOR2 (nuclear co-repressor) and coactivator (NCOA3), which were also included in FU Module 7. Their co-occurrence remain statistically significant with more stringent WeSCO test; the mutational spectrum was not decomposed due to small number of somatic mutations in the module. This suggests that the co-occurrence is less likely by chance and the mutations may jointly contribute to cancer progression.

Module 6 contains three long genes including MUC16 and TTN, for which recent studies caution that the frequent mutations might not necessarily be related to cancer progression even though they are often found significantly mutated in cancer [11]. The co-occurrence of TTN and MUC16 is not statistically significant when corrected with WeSCO for patient mutation frequency yet their co-occurrences with GON4L remain statistically significant (Table D in S1 Text). An interesting aspect of this cluster is the presence of APOBEC related signature (Signature 2) but no mismatch repair associated signatures. This might explain the co-occurrence of TTN and MUC16 and their mutual exclusivity with genes in other modules. While the mutations in TTN and MUC16 are suspected by many to be the results of mutagenic process rather than synergistic mutations in cancer, it is worth noting that MUC16 as well as GON4L can drive cancer growth when overexpressed [12,13]. While overexpression and mutations can have synergistic effects [14] and GON4L is found overexpressed in 26% of TCGA breast cancers, a mechanistic relation of these mutations in cancer progression remains still not clear.

Finally, we point out that the co-occurrence of gene pairs in modules 4 and 5 is not statistically significant when evaluated with WeSCO and thus is most likely related to sample mutation rates.

For BeME-WithCo, we used hypergeometric test to estimate the significance of co-occurrence. After obtaining the modules, we computed, for every pair of genes in each module, several measures of co-mutation including jaccard index and p-value for WeSCO test (Table D in S1 Text). Unlike hypergeometric test, WeSCO corrects for mutation frequency in each sample.

	gene1	gene2	jaccard index	#g1	#g2	hypergeometric	WeSCO
Module 1	DCC	TP53	0.041860465	12	212	0.002505756	0.03769
Module 2	MEF2A	NCOR2	0.1875	13	25	2.17E-06	4.20E-05
Module 2	NCOA3	MEF2A	0.24137931	23	13	3.34E-08	0
Module 2	NCOA3	NCOR2	0.263157895	23	25	6.52E-10	0
Module 3	BRCA2	RELN	0.107142857	12	19	0.003706016	0.0158
Module 3	BRCA2	PREX2	0.142857143	12	12	0.000904382	0.00425
Module 3	RELN	PREX2	0.107142857	19	12	0.003706016	0.0158
Module 4	PIK3CA	MAP2K4	0.072	237	31	0.0076195	0.218
Module 5	PCDH19	GATA3	0.060240964	13	75	0.009968893	0.0667
Module 6	GON4L	TTN	0.053571429	8	110	0.000382004	0.00485
Module 6	TTN	MUC16	0.12987013	110	64	0.001584299	0.135
Module 6	GON4L	MUC16	0.058823529	8	64	0.004077911	0.022923

Table D.Co-occurrence of genes within modules from BeME-WithCo. #g1 (#g2 respectively) is the number of samples in which gene1 (or gene2) in the pair has been mutated.

In Figure D in S1 Text, we show the mutated samples for each gene identified by BeME-WithCo setting.

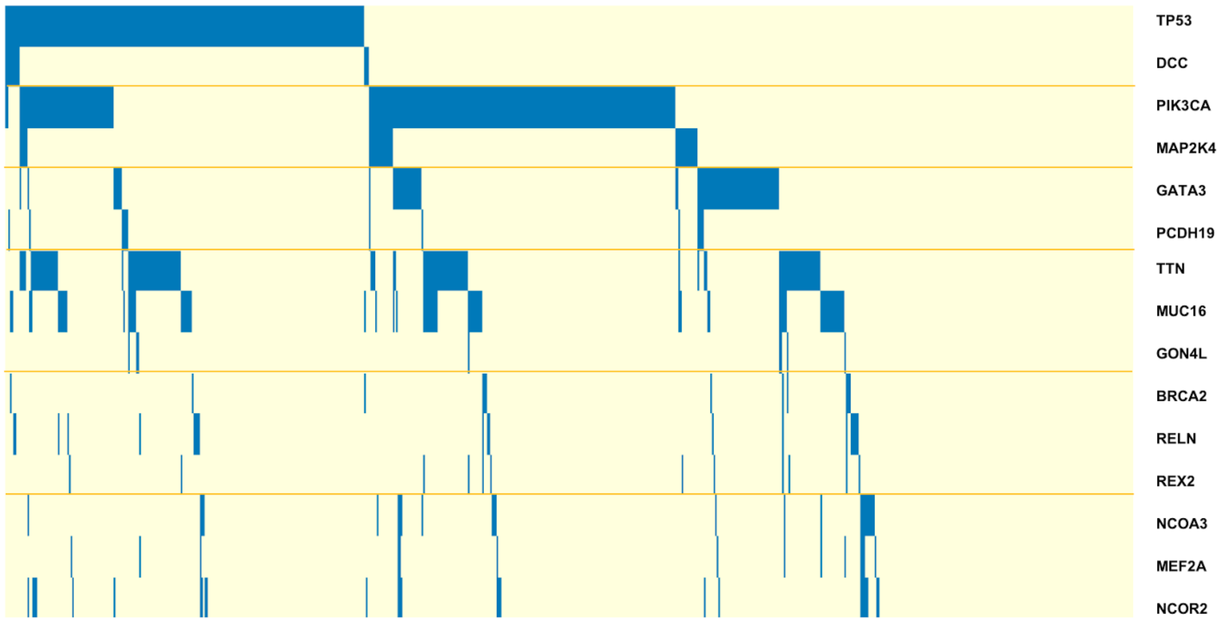

Fig. D. Mutated BRCA samples for each gene identified by BeME-WithCo

Additional information about BeCo-WithMEFun modules

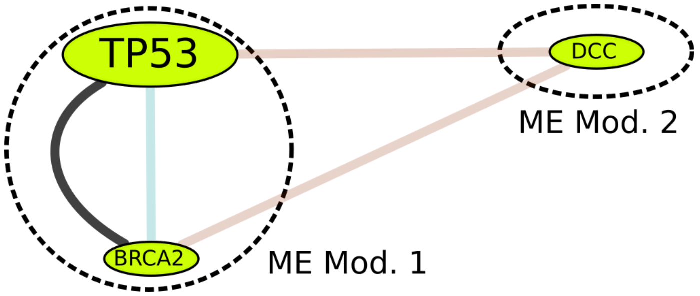

Fig. E. Modules uncovered by BeCo-WithMEFun for TCGA BRCA dataset. Edge color-coding and node size coding are the same as in Figure 2.

G. Application of BeWith to TCGA UCEC datasets

BeME-WithFun

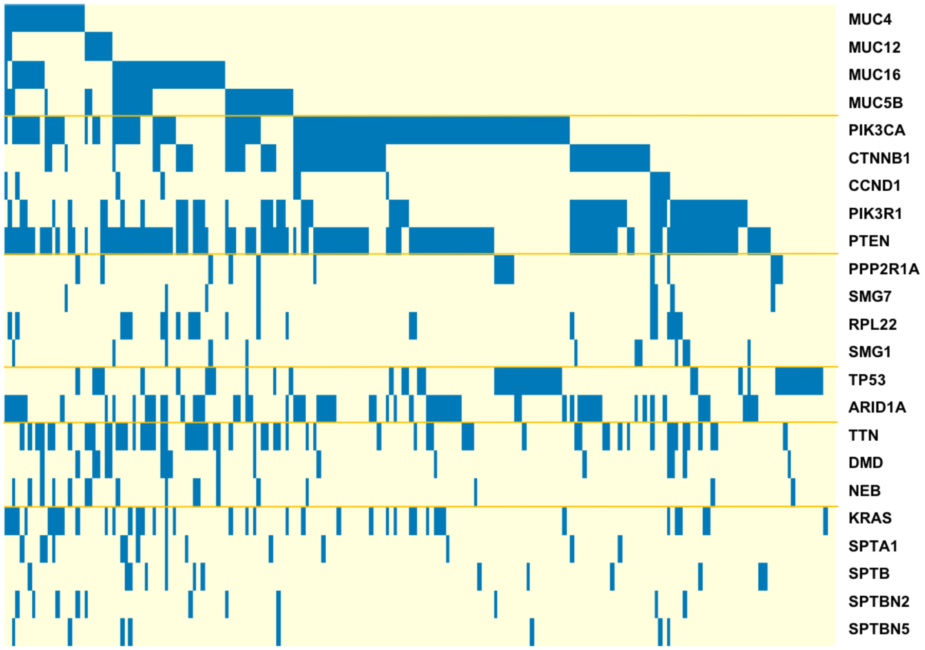

Fig F. Mutated UCEC samples for each gene identified by BeME-WithFun

BeME-WithCo

BeME-WithCo retrieved five modules including two one-element modules. Interestingly each of the modules contains at least one driver gene (Figure G in S1 Text).

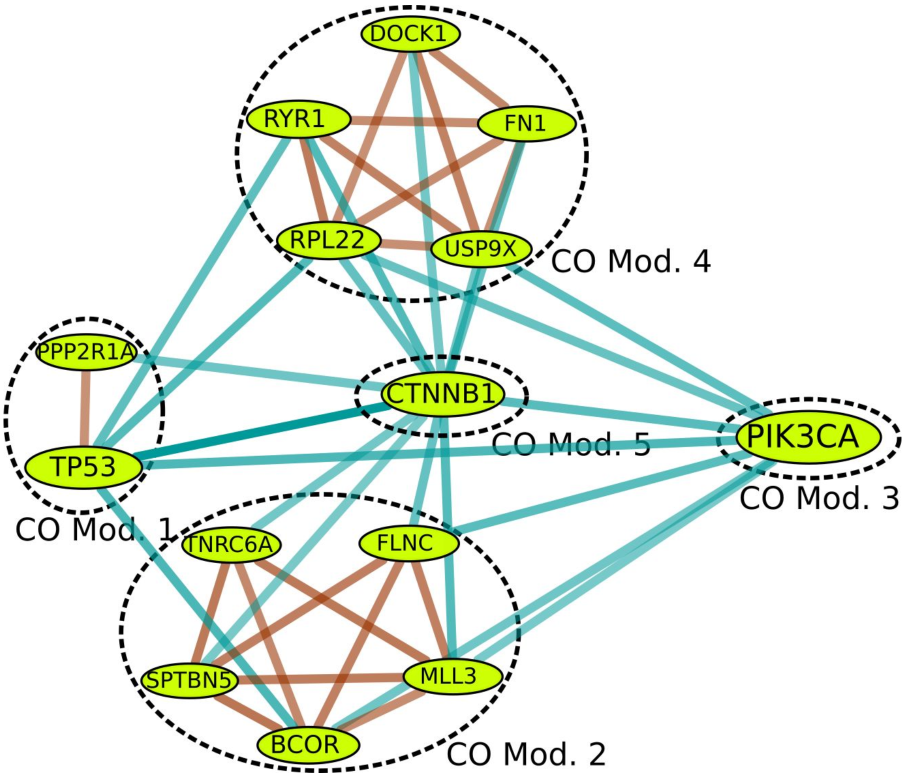

Fig G. Modules uncovered by BeME-WithCo for endometrial TCGA dataset. Edge color-coding and node size coding are the same as in Figure 2.

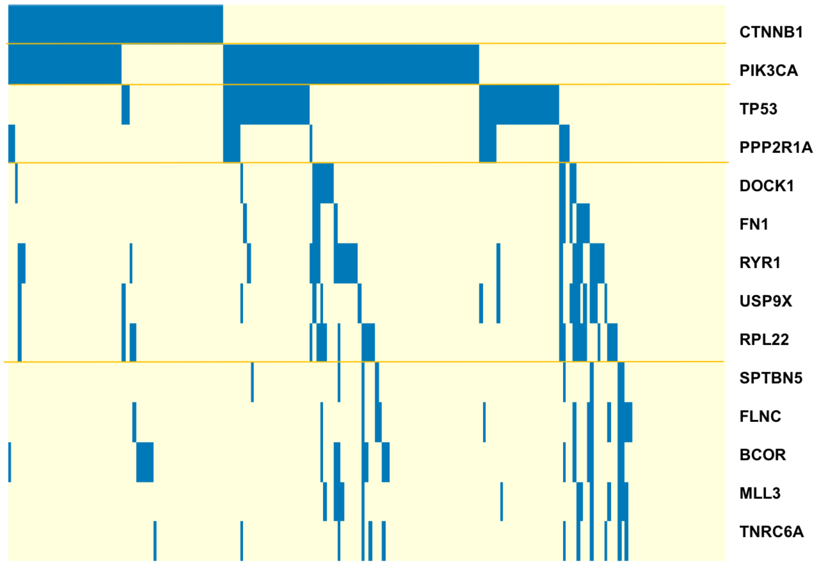

Fig H. Mutated UCEC samples for each gene identified by BeME-WithCo

BeCo-WithMEFun

BeCo-WithMEFun identified ATM gene as being co-mutated with an interacting pair containing RPL22 (Ribosomal protein L22) and SMG7 (one of nonsense mediated RNA decay genes). While these three genes have been linked to cancer [15], [16], [17], it remains to be established whether or not there is a synergistic relation between them.

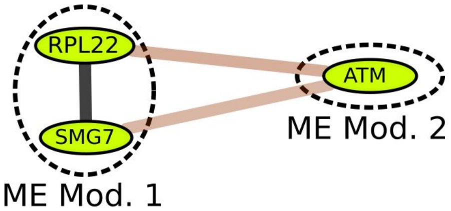

Fig I. Modules uncovered by BeCo-WithMEFun for TCGA UCEC dataset. Edge color-coding and node size coding are the same as in Figure 2.

H. BeWith Results on Different Number of Clusters:

We list the modules obtained by BeWith on BRCA and UCEC dataset with different number of clusters [K-1,K+1] where f(k)/k is maximized at K.

k	**Module List**	**# Drivers**	**Driver Enrichment Probability**	**Functional Distance**
5	FOXA1, GATA3<br>MAP2K4, MAP3K1<br>MUC4, MUC16, MUC5B, MUC12<br>PIK3CA, PTEN, TP53, AKT1<br>TTN, NEB, DMD	8	3.7e-4	1.0
6	FOXA1, GATA3<br>MAP2K4, MAP3K1<br>MUC4, MUC16, MUC5B, MUC12<br>PIK3CA, PTEN, TP53, AKT1<br>TTN, NEB, DMD<br>NCOA3, TBL1XR1, NCOR2, MED23	11	8.64e-6	1.0
7	FOXA1, GATA3<br>MAP2K4, MAP3K1<br>MUC4, MUC16, MUC5B, MUC12<br>TTN, NEB, DMD<br>NCOA3, TBL1XR1, NCOR2, MED23<br>MTOR, PTEN, TP53, AKT1, PIK3R1<br>PIK3CA, CDH1	14	6.90e-8	1.03

Table E-A BeME-WithFun on BRCA.

k	**Module List**	**# Drivers**	Driver Enrichment Probability

4	TP53, DCC<br>MUC16, TTN, GON4L<br>PIK3CA, MAP2K4<br>NCOA3, NCOR2, MEF2A	5	7.6e-3
5	TP53, DCC<br>MUC16, TTN, GON4L<br>PCDH19, GATA3<br>PIK3CA, MAP2K4<br>NCOA3, NCOR2, MEF2A	6	3.4e-3
6	TP53, DCC<br>MUC16, TTN, GON4L<br>PCDH19, GATA3<br>PIK3CA, MAP2K4<br>NCOA3, NCOR2, MEF2A<br>PREX2, RELN, BRCA2	7	2.4e-3

Table E-B. BeMEWithCo on BRCA.

k	**Module List**	**# Drivers**	**Driver Enrichment Probability**	**Functional Distance**
4	TP53, FBXW7<br>CCND1, CTNNB1, PIK3R1, PTEN, PIK3CA<br>MUC5B, MUC4, MUC12, MUC16<br>SMG7, PPP2R1A, SMG1, RPL22	9	1.35e-5	1.0
5	TP53, FBXW7<br>CCND1, CTNNB1, PIK3R1, PTEN, PIK3CA<br>DMD, TTN, NEB<br>MUC5B, MUC4, MUC12, MUC16<br>SMG7, PPP2R1A, SMG1, RPL22	9	9.15e-5	1.0
6	ARID1A, TP53<br>CCND1, CTNNB1, PIK3R1, PTEN, PIK3CA<br>DMD, TTN, NEB<br>MUC5B, MUC4, MUC12, MUC16<br>SMG7, PPP2R1A, SMG1, RPL22<br>SPTBN5, SPTB, KRAS, SPTBN2, SPTA1	10	1.53e-4	1.0

Table E-C BeME-WithFun on UCEC.

k	**Module List**	**# Drivers**	Driver Enrichment Probability
3	CTNNB1<br>SPTBN5, TNRC6A, MLL3<br>FN1, DOCK1, RPL22, USP9X, RYR1	3	0.07
4	CTNNB1<br>PPP2R1A, TP53<br>SPTBN5, BCOR, FLNC, TNRC6A, MLL3<br>FN1, DOCK1, RPL22, USP9X, RYR1	6	2.5e-3

5	CTNNB1<br>PIK3CA<br>PPP2R1A TP53<br>SPTBN5, BCOR, FLNC, TNRC6A, MLL3<br>FN1, DOCK1, RPL22, USP9X, RYR1	7	6.04e-4

Table E-D. BeMEWithCo on UCEC.

I. WeSME with BeME-WithFun modules

In column *module pv*, we compute the WeSME p-values between the genes and the corresponding modules while the columns *best pairwise p-value* contain the smallest WeSME p-values between the genes and any genes in the modules.

gene	module pv	best pairwise p-value
MED23	4.40E-05	0.000238
F8	0.000845	0.003535
FREM2	0.000845	0.003535
TBL1XR1	0.00199	0.00548
ATM	0.00215	0.01255
DNM3	0.00408	0.00922
STARD8	0.00408	0.00922
AOAH	0.0079	0.0084
BRWD3	0.00933	0.01817
KIF5A	0.00933	0.01817
LETM1	0.00933	0.01817
GPR112	0.00933	0.01817
ADD2	0.00933	0.01817
TRAPPC8	0.00933	0.01817
GRIK1	0.00933	0.01817
ZNF438	0.00933	0.01817
NUP214	0.00933	0.01817
MED15	0.00933	0.01817
DNAH1	0.01	0.0274
BRCA2	0.01	0.0274

Table F-A WeSME mutual exclusivity p-values between FU Module 1 (TP53, MTOR, PTEN, AKT1, PIK3R1) and other genes

gene	module pv	best pairwise p-value
USP36	7.00E-06	2.30E-05
PEG3	0.000169	0.000425
UTRN	0.00117	0.00278
CROCCP2	0.00119	0.00525
SPTA1	0.00143	0.0057
NWD1	0.00195	0.00366
DDR2	0.00195	0.00366
HECW2	0.00238	0.00573
VPS13C	0.002553	0.00573
DYNC2H1	0.00263	0.00586

RAB3GAP2	0.00408	0.00739
VCX3B	0.00408	0.00739
RBM23	0.00408	0.00739
DMBT1	0.00408	0.00739
MAGEC1	0.00408	0.00739
ITGA1	0.00408	0.00739
ZDBF2	0.00547	0.0114
BCORL1	0.00547	0.0114
NBPF1	0.00676	0.00821
COL6A5	0.00771	0.017147
DNAH8	0.0091	0.01776
DCC	0.0091	0.01776
ASXL3	0.0091	0.01776
SMCHD1	0.00975	0.0139
PRKCQ	0.00975	0.0139
AP1G2	0.00975	0.0139
CYP2A13	0.00975	0.0139
LETM1	0.00975	0.0139
GPR179	0.00975	0.0139
TNN	0.00975	0.0139
C20orf26	0.00975	0.0139
PARP8	0.00975	0.0139
MECOM	0.00975	0.0139
PTCHD2	0.00975	0.0139
ZNF687	0.00975	0.0139
TNIK	0.00975	0.0139
EWSR1	0.00975	0.0139
MED13	0.00975	0.0139
STAT4	0.00975	0.0139
AMPD1	0.00975	0.0139

Table F-B WeSME mutual exclusivity p-values between FU Module 2 (PIK3CA, CDH1) and other genes

gene	module pv	best pairwise p-value
ATM	0.0215	0.0341
GPR98	0.0215	0.0341
MDN1	0.024	0.0381
XIRP2	0.024	0.0381
PTEN	0.0211	0.0406
MUC4	0.0289	0.0482
DYNC2H1	0.0401	0.0551
NEB	0.0354	0.0704
DMD	0.0354	0.0704
COL12A1	0.0432	0.0756
PCNXL2	0.0432	0.0756
MED23	0.0432	0.0756
KIAA1109	0.0432	0.0756
UBR5	0.0432	0.0756
MUC17	0.0459	0.0822

Table F-C WeSME mutual exclusivity p-values between FU Module 3 (FOXA, GATA3) and other genes

gene	module pv	best pairwise p-value
MAP3K1	0	0.00003
GATA3	1.00E-06	0.000067
DCC	0.00677	0.0297
NCOA3	0.00718	0.009
RYR1	0.0122	0.015
NR1H2	0.0125	0.05165
CROCCP2	0.0223	0.0249
F8	0.0239	0.0675
MGAM	0.0285	0.0878
FLG	0.0365	0.13
WDR67	0.0384	0.0867
NFE2L3	0.0384	0.0867
RP1L1	0.0384	0.0867
MEFV	0.0384	0.0867
HRNR	0.0434	0.112

Table F-D WeSME mutual exclusivity p-values between FU Module 3 (TTN, DMD, NEB) and other genes

gene	module pv	best pairwise p-value
MAP3K1	0.000181	0.0213
PIK3CA	0.000454	0.00451
GATA3	0.000597	0.00549
PCDH15	0.00204	0.121
TSC22D1	0.0105	0.21
RUNX1	0.0163	0.0319
FAT2	0.0192	0.121
TBX3	0.0258	0.179
LRP1	0.0317	0.162
TEX15	0.0317	0.162
ODZ2	0.0317	0.162
MLLT4	0.0317	0.162
TG	0.0317	0.162
CSMD1	0.0371	0.122
HERC2	0.0391	0.168
MLL2	0.0391	0.0924
HCFC1	0.0495	0.178
ITPR1	0.0495	0.178
CDC42BPA	0.0495	0.178
COL14A1	0.0495	0.178

Table F-E WeSME mutual exclusivity p-values between FU Module 5 (MUC4, MUC16, MUC12, MUC5B) and other genes

gene	module pv	best pairwise p-value
FAT3	0.0381	0.15
DOCK11	0.0497	0.153
RB1	0.0497	0.153

HERC2	0.0497	0.153
CCDC66	0.0497	0.153
SHROOM4	0.0497	0.153
DNAH10	0.0255	0.111
DNAH3	0.0313	0.101
PIK3R1	0.0202	0.0843
PKHD1L1	0.0102	0.0576
DMD	0.0088	0.0476
MUC5B	0.0289	0.0662
HRNR	0.0289	0.0662
FLG	0.0292	0.059
RYR2	0.0188	0.0411
CDH1	0.0493	0.0675
MUC4	0.0278	0.0336
MUC16	0.0278	0.0336

Table F-F WeSME mutual exclusivity p-values between FU Module 6 (MAP3K1, MAP2K4) and other genes

gene	module pv	best pairwise p-value
TP53	1.30E-05	0.000282
MUC12	0.00105	0.0595
USH2A	0.00419	0.1001
RYR2	0.0241	0.109
NEB	0.0368	0.234
PKHD1L1	0.0377	0.242
MLL3	0.0437	0.142
FRG1B	0.044	0.127

Table F-G WeSME mutual exclusivity p-values between FU Module 7 (NCOR2, NCOA3, MED23, TBL1XR1) and other genes

REFERENCES

1. Kim Y-A, Madan S, Przytycka TM. WeSME: uncovering mutual exclusivity of cancer drivers and beyond. Bioinformatics [Internet]. 2016 May 3; Available from: http://dx.doi.org/10.1093/bioinformatics/btw242

2. Margot F. Exploiting orbits in symmetric ILP. Math Program. 2003;98(1-3):3–21.

3. Sherali HD, Cole Smith J. Improving Discrete Model Representations via Symmetry Considerations. Manage Sci. 2001;47(10):1396–407.

4. Denton BT, Miller AJ, Balasubramanian HJ, Huschka TR. Optimal Allocation of Surgery Blocks to Operating Rooms Under Uncertainty. Oper Res. 2010;58(4-part-1):802–16.

5. Milo R, Kashtan N, Itzkovitz S, Newman MEJ, Alon U. On the uniform generation of random graphs with prescribed degree sequences [Internet]. arXiv [cond-mat.stat-mech]. 2003. Available from: http://arxiv.org/abs/cond-mat/0312028

6. Leiserson MDM, Blokh D, Sharan R, Raphael BJ. Simultaneous identification of multiple driver pathways in cancer. PLoS Comput Biol. 2013 May 23;9(5):e1003054.

7. Kim Y-A, Cho D-Y, Dao P, Przytycka TM. MEMCover: integrated analysis of mutual exclusivity and functional network reveals dysregulated pathways across multiple cancer types. Bioinformatics. 2015 Jun 15;31(12):i284–92.

8. Zhang J, Junhua Z, Ling-Yun W, Xiang-Sun Z, Shihua Z. Discovery of co-occurring driver pathways in cancer. BMC Bioinformatics. 2014;15(1):271.

9. Rosenthal R, McGranahan N, Herrero J, Taylor BS, Swanton C. DeconstructSigs: delineating mutational processes in single tumors distinguishes DNA repair deficiencies and patterns of carcinoma evolution. Genome Biol. 2016 Feb 22;17:31.

10. Nik-Zainal S, Davies H, Staaf J, Ramakrishna M, Glodzik D, Zou X, et al. Landscape of somatic mutations in 560 breast cancer whole-genome sequences. Nature. 2016 May 2;534(7605):47–54.

11. Lawrence MS, Stojanov P, Polak P, Kryukov GV, Cibulskis K, Sivachenko A, et al. Mutational heterogeneity in cancer and the search for new cancer-associated genes. Nature. 2013 Jul 11;499(7457):214–8.

12. Lakshmanan I, Ponnusamy MP, Das S, Chakraborty S, Haridas D, Mukhopadhyay P, et al. MUC16 induced rapid G2/M transition via interactions with JAK2 for increased proliferation and anti-apoptosis in breast cancer cells. Oncogene. 2012 Feb 16;31(7):805–17.

13. Agarwal N, Dancik GM, Goodspeed A, Costello JC, Owens C, Duex JE, et al. GON4L Drives Cancer Growth through a YY1-Androgen Receptor-CD24 Axis. Cancer Res. 2016 Sep 1;76(17):5175–85.

14. Berger MF, Hodis E, Heffernan TP, Deribe YL, Lawrence MS, Protopopov A, et al. Melanoma genome sequencing reveals frequent PREX2 mutations. Nature. 2012 May 9;485(7399):502–6.

15. Ahmed M, Rahman N. ATM and breast cancer susceptibility. Oncogene. 2006;25(43):5906–11.

16. Luo H, Hongwei L, Lauren C, Guowu Y, Wenguo J, Yi T. SMG7 is a critical regulator of p53 stability and function in DNA damage stress response. Cell Discovery. 2016;2:15042.

17. Kandoth C, Schultz N, Cherniack AD, Akbani R, Liu Y, Shen H, et al. Integrated genomic characterization of endometrial carcinoma. Nature. 2013 May 2;497(7447):67–73.
